# Medication self-management interventions for persons with stroke: A scoping review

**DOI:** 10.1371/journal.pone.0285483

**Published:** 2023-05-18

**Authors:** Lauren Cadel, Stephanie R. Cimino, Glyneva Bradley-Ridout, Sander L. Hitzig, Tejal Patel, Chester H. Ho, Tanya L. Packer, Aisha K. Lofters, Shoshana Hahn-Goldberg, Lisa M. McCarthy, Sara J. T. Guilcher

**Affiliations:** 1 Leslie Dan Faculty of Pharmacy, University of Toronto, Toronto, ON, Canada; 2 Institute for Better Health, Trillium Health Partners, Mississauga, ON, Canada; 3 Rehabilitation Sciences Institute, Temerty Faculty of Medicine, University of Toronto, Toronto, ON, Canada; 4 St. John’s Rehab Research Program, Sunnybrook Research Institute, Sunnybrook Health Sciences Centre, Toronto, ON, Canada; 5 Gerstein Science Information Centre, University of Toronto, Toronto, ON, Canada; 6 Department of Occupational Science and Occupational Therapy, Temerty Faculty of Medicine, University of Toronto, Toronto, ON, Canada; 7 University of Waterloo School of Pharmacy, Kitchener, ON, Canada; 8 Schlegel-University of Waterloo Research Institute of Aging, Waterloo, ON, Canada; 9 Division of Physical Medicine & Rehabilitation, Department of Medicine, University of Alberta, Edmonton, AB, Canada; 10 Schools of Occupational Therapy and Health Administration, Dalhousie University, Halifax, NS, Canada; 11 Department of Rehabilitation, Radboud University Medical Centre, Nijmegen, The Netherlands; 12 Department of Family and Community Medicine, University of Toronto, Toronto, ON, Canada; 13 Women’s College Research Institute, Toronto, ON, Canada; 14 OpenLab, University Health Network, Toronto, ON, Canada; 15 Institute of Health Policy, Management and Evaluation, University of Toronto, Toronto, ON, Canada; The Hong Kong Polytechnic University, HONG KONG

## Abstract

The use of multiple medications is common following a stroke for secondary prevention and management of co-occurring chronic conditions. Given the use of multiple medications post-stroke, optimizing medication self-management for this population is important. The objective of this scoping review was to identify and summarize what has been reported in the literature on interventions related to medication self-management for adults (aged 18+) with stroke. Electronic databases (Ovid Medline, Ovid Embase, EBSCO CINAHL, Ovid PsycINFO, Web of Science) and grey literature were searched to identify relevant articles. For inclusion, articles were required to include an adult population with stroke undergoing an intervention aimed at modifying or improving medication management that incorporated a component of self-management. Two independent reviewers screened the articles for inclusion. Data were extracted and summarized using descriptive content analysis. Of the 56 articles that met the inclusion criteria, the focus of most interventions was on improvement of secondary stroke prevention through risk factor management and lifestyle modifications. The majority of studies included medication self-management as a component of a broader intervention. Most interventions used both face-to-face interactions and technology for delivery. Behavioural outcomes, specifically medication adherence, were the most commonly targeted outcomes across the interventions. However, the majority of interventions did not specifically or holistically target medication self-management. There is an opportunity to better support medication self-management post-stroke by ensuring interventions are delivered across sectors or in the community, developing an understanding of the optimal frequency and duration of delivery, and qualitatively exploring experiences with the interventions to ensure ongoing improvement.

## Introduction

Stroke is one of the leading causes of morbidity and mortality worldwide [1]. Globally in 2019, approximately 12.2 million strokes and 6.6 million stroke-related deaths occurred [1]. Common risk factors for stroke include older age, physical inactivity, consumption of alcohol, poor diet, lower socioeconomic status, and comorbidities such as hypertension, coronary heart disease, diabetes, depression, chronic pain, and atrial fibrillation [1–5]. Individuals who have experienced a stroke have a higher prevalence of comorbidities (43% - 94% of the population) than those without stroke [4, 6, 7].

Medications are commonly used for the prevention of secondary strokes (ischemic and hemorrhagic), as well as for the management of comorbidities and secondary conditions related to the stroke itself [4, 8]. Importantly, adherence to medication post-stroke is critical for minimizing the risk of recurrent strokes [9]. However, medication adherence may be a precursor to the effectiveness of these medication regimens and can be impacted by a multitude of factors, such as an individuals’ beliefs and concerns about medications, functional ability, and access to medication, including who prescribes the medication and how and where it is dispensed [10–12].

Rates of polypharmacy (use of multiple medications, often five or more) vary among adults who experienced a stroke, but have been reported to range between 26% and 75% [4, 13–15]. Despite the potential need for multiple medications to prevent secondary strokes and manage comorbidities (e.g., hypertension, coronary heart disease, diabetes, depression) and secondary conditions (e.g., chronic pain), there is also the risk of potentially inappropriate medication use (e.g., medications with high risk of adverse events) [13]. For example, Matsumoto and colleagues conducted a retrospective cohort study of 361 older adults (aged 65+) who had experienced a stroke and identified potentially inappropriate medications in 65% of patients at discharge [13]. Common potentially inappropriate medications prescribed at discharge included antipsychotics, benzodiazepines, proton pump inhibitors, and non-steroidal anti-inflammatory drugs [13]. Among individuals who have experienced a stroke, polypharmacy has been associated with reduced functional and rehabilitative outcomes [13–15].

Polypharmacy often increases the complexity of a medication regimen [16], which can impact medication-self-management. Individuals with stroke have reported challenges with their overall medication management, including understanding, medication-taking self-efficacy, and medication burden [17–20]. Medication self-management can be impacted by patient, provider, and system level factors [21], including, but not limited to, the number of medications prescribed, complexity of medication regimens, receipt of medication education/ instructions, cost, medication-related supports (e.g., pillboxes and blister packs), healthcare provider knowledge, access to interventions, and structure of the healthcare system [4, 22, 23]. Further to this, a stroke may result in residual deficits that impact an individual’s ability to self-manage their medications.

Optimal medication management, including medication self-management, following a stroke is important to reduce the risk of a recurrent stroke. For this scoping review, we define medication self-management as the range of tasks, skills, and behaviours associated with an individual’s capability, opportunity, and motivation to navigate the physical, social, and cognitive lifestyle factors, changes, and consequences inherent in taking, or choosing not to take, medications in everyday life. These tasks include having the knowledge and related confidence to deal with medical, emotional, and role management, as well as the core skills of problem-solving, decision-making, seeking formal and informal supports, self-tailoring, goal-setting, optimizing social interactions, and engaging in activities, as they relate to managing medications [24, 25].

Given the evidence demonstrating that people who experienced a stroke are prescribed inappropriate medications, along with the data showing the challenges people experience with managing their medications (knowledge, adherence, access), more work is needed to understand how to better support this population with medication self-management. Therefore, the purpose of this scoping review was to identify and summarize what was reported in the literature on interventions related to medication self-management for adults with stroke. Specific aims included identifying the content of the interventions, how the interventions were designed and delivered, the outcome measures used to evaluate the interventions, and the impact of the interventions.

## Methods

We conducted this scoping review in accordance with the methodology outlined by Peters et al. [26]. The reporting guidelines for scoping reviews, Preferred Reporting Items for Systematic Reviews and Meta-Analyses–Scoping Review Extension (PRISMA-ScR), were also followed (see S1 Table) [27].

### Protocol and registration

The scoping review protocol was registered on Open Science Framework (https://osf.io/89qha).

### Eligibility criteria

This scoping review was our second knowledge synthesis related to medication self-management, as part of a larger research study that aims to develop and evaluate a toolkit for medication self-management for persons with spinal cord injury. The first scoping review examined literature related to medication self-management for persons with spinal cord injury [28]. Given that there was limited research within spinal cord injury, we expanded the eligible population to also include persons who have experienced stroke (neurological population that often experiences life-altering changes and significant change to medication regimen). The findings of these reviews are being presented separately based on fundamental differences noted during analysis, such as the state of the research, the content of the interventions, and the outcomes measured.

The inclusion criteria for articles for this review were: (1) an adult population (majority aged 18+) that experienced a stroke; (2) an intervention aimed at modifying or improving medication management; and (3) the intervention incorporated a component of self-management. To meet the first inclusion criteria, at least 50% of the participants had to be over the age of 18 with stroke. We limited this review to adults because the tasks and skills related to medication self-management for a youth population would likely be structured and delivered in a different manner. For the second criteria, medication management was defined as the tasks, skills, and behaviours associated with an individual’s capability, opportunity, and motivation to navigate the physical, social, and cognitive lifestyle factors, changes, and consequences inherent in taking, or choosing not to take, medications. For the third criteria, the intervention had to include a component of self-management. This was based our definition for this scoping review described above. The exclusion criteria were: (4) opinion pieces; (5) conference abstracts; (6) study protocols; or (7) the inability to access the full-text. We excluded conference abstracts and study protocols to ensure all included articles presented finalized results to fully examine their implementation characteristics and associated outcomes. We also excluded knowledge syntheses from data extraction, but reviewed their reference lists for potential articles.

### Search methods

An academic health sciences librarian (GBR) developed the search strategy through frequent consultations with members of the research team (LC and SJTG). The following five electronic databases were searched on March 11th, 2022: MEDLINE (Ovid Interface), EMBASE (Ovid Interface), CINAHL Plus (EBSCOhost Interface), APA PsycINFO (Ovid Interface), and Clarivate Web of Science. A second academic librarian PRESS peer-reviewed the Ovid MEDLINE search prior to search translations [25]. The concepts of (traumatic spinal cord injury OR stroke) AND self management AND medication were combined to develop the search. Using each platforms’ command language and controlled vocabulary, the search was translated into the different databases, without limits. The full database search strategies can be found in S2 Table. Grey literature was searched on July 26, 2022. Websites and repositories (World Health Organization, Heart and Stroke Foundation, American Stroke Association, University of Toronto TSpace) were searched for relevant articles.

### Selection process

We followed Bramer’s method for deduplication using EndNote X8 [29]. Articles were uploaded to a knowledge synthesis software platform, Covidence, for screening. An interrater test of the titles and abstracts of 150 articles was conducted by three screeners (LC, SRC, SJTG) to ensure good agreement and interpretation of the eligibility criteria. No revisions were made to the eligibility criteria following the interrater test as the screeners had an agreement of over 95%. The remaining titles and abstracts were independently screened by two reviewers and any disagreements that occurred were resolved through consensus. Two screeners (LC and SRC) completed an interrater test of 10 full-text articles to ensure good agreement. With 90% agreement, the remaining full-text articles were double screened by the same screeners. All disagreements were resolved through consensus. If the full-text was published in a language other than English, it was translated using Google Translate and the article was screened using the translated version.

### Data charting process

A study-specific, data extraction table was developed in Microsoft Excel. The data extraction table was tested by two extractors (LC and SRC) and no revisions were made. These team members also reviewed each others’ first article extraction to ensure all data was extracted accurately and consistently. Following this review, data were independently extracted from the remaining articles by a single extractor (LC, SRC).

### Data items

The data extraction process involved collating information related to the article (title, authors, year of publication, journal, funding), study description (objective, type of population, method of data collection, study design, theoretical orientation, eligibility criteria, outcomes, country, setting), intervention (description, content, frequency, duration, single or multi-component, format, tailoring, modifications, method of delivery, setting), population (sample size, age, sex, gender, ethnicity/race, income, education, marital status, household composition, employment status, comorbidities), outcomes and findings (results and key findings, conclusions). The Template for Intervention Description and Replication (TIDieR) checklist was used to inform the intervention information that was extracted [30].

### Synthesis methods

Descriptive approaches were used to synthesize the extracted data. More specifically, descriptions of the study designs, countries, years of publication, intervention characteristics, and intervention outcomes are provided. To synthesize the intervention outcomes, one team member (LC) further categorized them into the following: learning outcomes (knowledge, skills, abilities, attitudes, and understanding achieved through participation in the intervention [31]), behavioural outcomes (actions that individuals consciously engaged or did not engage in [32]), or clinical outcomes (changes in health, function, or quality of life [33]). We used the TIDieR checklist to guide the presentation of the results [30]. A critical appraisal of articles was not conducted and it is not a requirement of scoping reviews [27].

## Results

### Study selection

**Fig 1:**
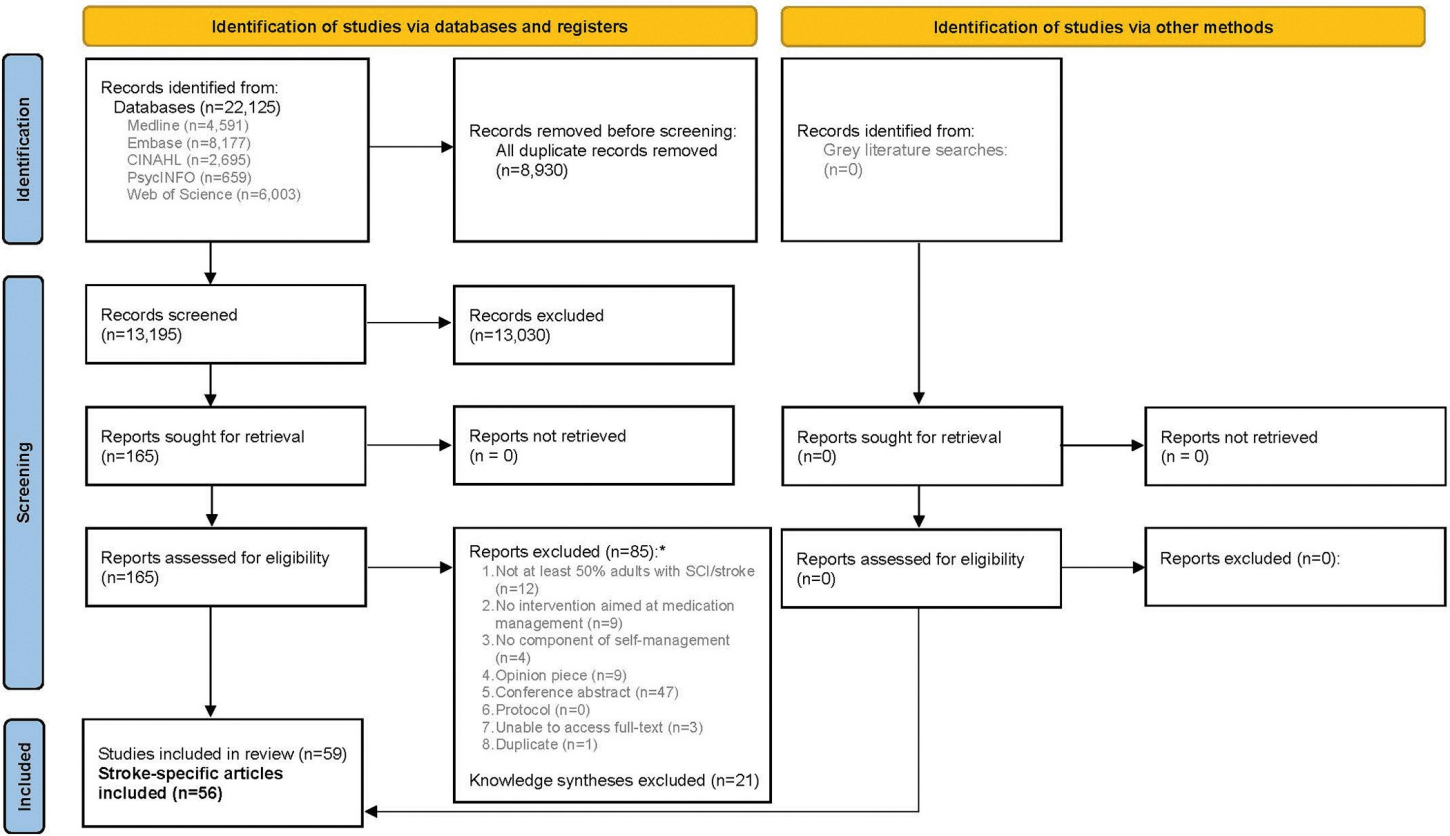
PRISMA 2020 flow diagram of included articles.

The database searches identified 22,125 articles (see Fig 1). Following deduplication, 13,195 articles remained for title and abstract screening. During title and abstract screening, 13,030 articles were excluded, leaving 165 articles for the full-text screen. During full-text screening, 85 articles were excluded. Knowledge syntheses (n = 21) were reviewed for relevant articles, but not included in data extraction or analysis, resulting in 59 relevant articles. For this scoping review, we only present the results of the stroke articles, which resulted in 56 included articles.

### Study characteristics

Characteristics of included articles are displayed in Table 1. The majority of the included studies used a quantitative study design (n = 53) [34–86]. There were two mixed methods studies [87, 88] and one qualitative study [89]. The most common quantitative study designs included randomized controlled trials (n = 31) and prospective studies (n = 11). Most articles (n = 53) were published after 2010 [34–61, 63–66, 68–70, 72–89], with only three being published prior [62, 67, 71]. Studies were conducted across 20 different countries, with the majority conducted in the United States (n = 14) [38, 42–45, 50, 57, 59, 67, 74, 81–83, 88] and China (n = 11) [40, 46, 73, 76–80, 84–86]. Other countries included the United Kingdom (n = 4) [63, 64, 69, 72], South Korea (n = 2) [55, 56], Australia (n = 2) [41, 65], Belgium (n = 2) [54, 75], Germany (n = 2) [49, 60], Ghana (n = 2) [68, 89], Hong Kong (n = 2) [70, 71], India (n = 2) [34, 39], Malaysia (n = 2) [35, 87], New Zealand (n = 2) [36, 61], Pakistan (n = 2) [52, 53], Austria (n = 1) [47], Denmark (n = 1) [48], France (n = 1) [37], Israel (n = 1) [62], Japan (n = 1) [66], Thailand (n = 1) [58], and Turkey (n = 1) [51].

**Table 1:** Characteristics of included articles (n = 56).

Author (year), Country	Objective	Study Design	Participants	Sample Size	Primary Outcome Measures	Key Findings
Annie et al. (2021), India [34]	• To explore the impact of clinical pharmacist interventions on the prevention of secondary strokes • To examine prescription medication use for secondary stroke prevention	Prospective interventional study	Stroke (aged 18+, admitted to and discharged from stroke unit)	100	• Stroke knowledge and awareness • Medication adherence (Morisky Medication Adherence 8- item scale) • Direct medical cost for treatment Risk of future stroke (CHADS_2_ Score and CHA_2_ DS_2_ -VASc score)	• Clinical pharmacists can contribute to secondary stroke prevention through enhanced knowledge and improved adherence
Appalasamy et al. (2020), Malaysia [87]	• To explore the feasibility and acceptability of a video narrative intervention post-stroke • To examine the impact of the intervention on medication understanding and use self-efficacy and blood pressure control	Randomized controlled trial and semi-structured interviews	Stroke (first diagnosed stroke within 6 months and prescribed stroke risk prevention medications)	54	• Medication understanding and use self-efficacy (MUSE)	• Medication use self-efficacy improved in the intervention and control groups, with greater improvements seen in the intervention group • Learnings about recruitment, feasibility, and acceptability will be applied to a future study
Appalasamy et al. (2020), Malaysia [35]	• To explore the effectiveness of a post-stroke video narrative intervention on medication understanding and use self-efficacy and associated factors	Randomized controlled trial (single blind)	Stroke (aged 18+, diagnosis of stroke within 6 months, satisfactory literacy, prescribed antithrombotic & antihypertensive medication)	216	• Medication understanding and use self-efficacy (MUSE)	• Personalized video narratives motivated participants and improved medication use self-efficacy
Barker-Collo et al. (2015), New Zealand [36]	• To examine the effectiveness of Motivational Interviewing on medication (blood pressure and cholesterol lowering) adherence	Randomized controlled trial (single blind, prospective phase III)	Stroke (aged 16+)	386	• Systolic blood pressure and low density lipoprotein cholesterol levels	• Medication adherence was significantly improved with motivational interviewing
Ben Nasr et al. (2018), France [37]	• To assess the effects of an educational program on knowledge, disease management, medication adherence and blood pressure self-management	Prospective recruitment cohort	Hypertensive stroke patients	64	• Patient knowledge and skills • Patient disease management (medication adherence and blood pressure) • Patient satisfaction	• Knowledge, medication adherence, and blood pressure self-management can be improved through an educational program
Bretz et al. (2014), United States [38]	• To explore the impact of an education-based intervention on medication adherence and lifestyle modification	Prospective study	Ischemic Stroke	193	• Health status (Short-Form Health Survey (SF-12))	• The education-based intervention resulted in reduced rehospitalization, increased medication adherence, and high patient satisfaction
Chandrasekhar et al. (2018), India [39]	• To examine the impact of Pharmaceutical Care on health-related quality of life	Prospective interventional study	Stroke	128	• Health-related quality of life (Short form [SF]-36) • Medication adherence (Morisky Medication Adherence Scales MMAS-8)	• Health-related quality of life and medication adherence were positively impacted • Pharmaceutical care provided in hospital was feasible
Chen et al. (2016), China [40]	• To evaluate the effects of a Home Care model on length of hospital stay, satisfaction with acute stay, and rates of medication compliance, complications and readmission	Quasi-experimental evaluation	Cerebral Stroke	341	• Average length of hospital stay • Satisfaction with acute hospitalisation Ability to perform activities of daily living (Barthel Index) • Rates of stroke recurrence • Self-reported medication compliance • Aspiration pneumonia • Stroke-related readmission	• The home care model positively impacted medication compliance, ability to perform activities of daily living, quality of life, length of stay, and readmission rates
Coombes et al. (2020), Australia [41]	• To assess the feasibility of a Patient-Centred Educational Exchange on shared conversation about stroke prevention medications	Non-comparative, prospective study	Stroke (aged 18+, managed own medication, planned to be discharged home)	16	• Perceptions about stroke (Brief‐Illness Perceptions Questionnaire) • Perceptions about medications (Beliefs about Medicines Questionnaire‐specific) Medication‐taking behaviour (Morisky‐Green‐Levine adherence questionnaire)	• The educational exchange was feasible to implement and allowed participants to be engaged in sharing their perceptions and beliefs
Damush et al. (2016), United States [42]	• To assess the impact of a Veterans Stroke Prevention Program on self-efficacy, stroke self-management practices, medication adherence, and stroke-specific, health-related quality of life	Randomized controlled trial (pilot)	Ischemic Stroke or Transient Ischemic Attack (aged 18+, acute diagnosis of stroke within 12 months)	174	• Patient self-efficacy • Stroke-specific, health-related quality of life • Medication adherence	Medication adherence improved when receiving the post-stroke, self-management prevention program
Deen et al. (2018), United States [43]	• To explore the impact of a stroke nurse navigation program on concurrent chart reviews and patient compliance	Chart review and observational longitudinal study	Ischemic Stroke (aged 18–90, discharged home or to rehabilitation hospital)	Phase 1: Not clearly reported Phase 2: 100	• Medication adherence • Physician follow-up adherence • Emergency department visits • Smoking • Functional status (Barthel Index) • Quality of life (EQ-5D Quality of Life Scale) • Readmissions	• Stroke nurse navigation had positive impacts on medication compliance, follow-up appointments, emergency department visits, and readmissions
Duncan et al. (2020), United States [44]	• To develop and assess the effectiveness of a Comprehensive Post Acute Stroke Services Transitional Care management program	Cluster-randomized pragmatic trial	Ischemic or Hemorrhagic Stroke or Transient Ischemic Attack (aged 18+, discharged home)	5,882	• Functional status (Stroke Impact Scale-16)	• The transitional care program did not significantly affect functional status
Evans-Hudnall et al. (2014), United States [45]	• To assess the impact of a multi-behavioural stroke self-care program for underserved racial/ethnic minority groups on stroke knowledge, treatment effects, health behaviours, depression, and anxiety	Randomized, two-group experimental design	Stroke, Transient Ischemic Attack or Ischemic Attack (aged 18+, going to be discharged home)	52	• Stroke knowledge (US Behavioral Surveillance Survey) • Exercise (US Behavioral Surveillance Survey) • Fruit and vegetable consumption (US Behavioral Surveillance Survey) • Tobacco and alcohol use (US Behavioral Surveillance Survey) • Medication adherence (US Behavioral Surveillance Survey)	• Secondary stroke risk factors reduced recurrent risk factors and improved stroke knowledge
Feng et al. (2021), China [46]	• To assess the impact of the Hospital-Community Integrated Service Model on self-care ability, compliance, self-efficacy and mood	Randomized controlled trial	Stroke (aged 60+)	120	• Self-care ability (modified Barthel Index) • Compliance behavior Self-efficacy (General Self-efficacy Scale) • Adverse mood (Zung’s Self-rating Anxiety Scale and Zung’s Self-rating Depression Scale)	• The integrated service model improved self-care ability, self-efficacy, medical compliance, and reduced negative emotions
Fruhwirth et al. (2022), Austria [47]	• To examine the effectiveness of a smartphone app on risk factor management for secondary stroke prevention	Pilot prospective study with pre-post intervention design	Ischemic stroke (aged 18–55)	42	• Modifiable stroke risk factors (physical activity, nutrition, alcohol consumption, smoking behaviour, obesity, and hypertension)	• A smartphone app intervention can promote motivation, increase physical activity, and improve quality of life
Hedegaard et al. (2014), Denmark [48]	• To evaluate a pharmacist-led, multi-faceted intervention on medication adherence for secondary stroke prevention	Randomized controlled trial	Ischemic Stroke or Transient Ischemic Attack (aged 18+, stroke within 30 days, prescribed at least 1 antiplatelet or anticoagulant medication)	203	• Adherence to the thrombopreventive regimen (composite medication possession ratio)	• Medication adherence, persistence to secondary stroke prevention, and clinical outcomes were not impacted by the pharmacist-led, multi-faceted intervention
Hohmann et al. (2013), Germany [49]	• To evaluate the impact of a clinical pharmacist-led intervention on adherence to hospital discharge medication	2-Phase Prospective, Interventional Study	Ischemic Stroke or Transient Ischemic Attack (aged 18+, taking 2+ drugs during hospital stay, discharged from hospital)	312	• Adherence to hospital discharge medication	• Medication adherence can be improved by providing information and education on new medications and medication changes
Holzemer et al. (2011), United States [50]	• To examine the impact of intensive, individualized educational plans on attention to risk factors and lifestyle changes	Randomized controlled trial	Ischemic Stroke or Transient Ischemic Attack (aged 18–89)	Enrolled: 52 Completed: 27	• Risk factor management (medication compliance, smoking, diet and exercise, blood pressure, body mass index, and cholesterol)	• The individualized educational plans significantly improved adherence to risk factor modification
Kalav et al. (2022), Turkey [51]	• To assess the effect of an intervention based on the chronic care model on self-management, quality of life, and patient satisfaction	Randomized controlled trial (single blind)	Ischemic Stroke (aged 18+)	68	• Self-efficacy (Stroke Self-Efficacy Questionnaire) • Quality of life (Stroke Specific Quality of Life Scale) • Patient satisfaction (Patient Assessment of Chronic Illness Care)	• No significant effects on self-efficacy, quality of life, or activities of daily living were seen, but satisfaction level increased
Kamal et al. (2018), Pakistan [52]	• To develop and test an individualized health information technology intervention on medication adherence	Randomized controlled trial (single blind)	Cerebrovascular Accident or Coronary Artery Disease (aged 18+, use of anti-platelets and/or statins	197	• Medication adherence (8-item Morisky Medication Adherence scale 8)	• No significant effects were identified for medication adherence, but was found to be feasible
Kamal et al. (2015), Pakistan [53]	• To explore the effectiveness of a tailored Short Messaging Service on medication adherence and blood pressure	Randomized controlled superiority trial (single blind)	Stroke (aged 19+, at least 1 month post-stroke, use of 2+ drugs)	200	• Medication adherence (Urdu version of Morisky Medication Adherence Questionnaire)	• The tailored Short Messaging Service improved medication adherence and blood pressure management
Kamoen et al. (2020), Belgium [54]	• To test the impact of a personal digital coaching program on cardiovascular risk control post-stroke	Prospective, multicenter, interventional cohort study	Ischemic Stroke (aged 18+, hospitalized with stroke at least 2 weeks prior, expected to be discharged home)	216	• Cardiovascular risk (Systematic COronary Risk Evaluation: High and Low cardiovascular Risk Chart)	• Rates of stroke recurrence rates reduced and adherence and quality of life improved following the coaching program
Kim and Park (2011), Korea [55]	• To develop and test a web-based secondary prevention educational program on knowledge, family support, health behaviour performance, and health status	Prospective non-randomized intervention study	Ischemic Stroke (hypertension, smoked at admission)	50	• Knowledge (disease-related knowledge measurement tool) • Family support (family support behaviour measurement too) • Health behavior performance (health behaviour performance measurement tool) • Health status (modified three-item tool)	• The web-based, educational program improved knowledge of patients and families, family support, and health behaviour compliance
Kim et al. (2020), Korea [56]	• To develop an mHealth platform to facilitate post-stroke care • To examine the impact of the mHealth platform on stroke awareness, mood, and quality of life, adherence to app use, satisfaction, blood pressure control, and physical measurements	Prospective, non-randomized, interventional study	Ischemic and Hemorrhagic Stroke (aged 19+)	99	• System Utilization • System Satisfaction • Stroke Awareness • Depression (Beck Depression Inventory-II) • Health-Related Quality of Life (EuroQol-5 Dimensions) • Physical Measurements	• The mHealth system improved stroke awareness, depression, and blood pressure control
Kitzman et al. (2017), United States [57]	• To assess the community navigation needs post-stroke to facilitate transitions back to rural, low-resource communities and improve health outcomes	Pilot program assessment	Stroke (from low resource, rural communities)	30	• Number and types of stroke related risk factors • Type of follow-up education provided • Types of resources accessed (enrolling in healthcare plans, medications waiver program, durable medical equipment, home modifications, etc.) • Number of 30-day hospital readmissions and emergency department visits • Compliance with medications, physician visits, outpatient rehabilitation visits	• Community navigation post-stroke was effective in supporting transitions back to rural communities
Komton (2021), Thailand [58]	• The examine the effectiveness of the SystemCHANGE™ intervention on healthy eating, physical activity, and medication adherence	Randomized controlled trial	Ischemic and Hemorrhagic Stroke (aged 18–80, discharged to own home or family’s home, living in Bangkok Metropolitan Region)	108	• Healthy eating (Food Frequency Questionnaire) • Physical activity (International Physical Activity Questionnaire Short Form) • Medication adherence (Thai version of 8-item Morisky Medication Adherence Scale)	• SystemCHANGE™ promoted health behaviour change in physical activity, medication adherence, and healthy eating
Kronish et al. (2014), United States [59]	• To develop a culturally-tailored, peer-led stroke prevention workshop To assess the impact of the workshop on blood pressure control, low-density lipoprotein cholesterol, and antithrombotic use	Randomized controlled trial	Stroke (aged 40+ and stroke within past 5 years)	600	• Proportion of participants who achieved goals for the 3 stroke prevention measures: blood pressure, low density lipoprotein cholesterol, and antithrombotic use	• The peer-led stroke prevention workshop supported improved blood pressure, but did not impact low-density lipoprotein cholesterol or antithrombotic use
Leistner et al. (2012), Germany [60]	To examine the quality of secondary stroke prevention To develop a support program that improves risk factor control and medication adherence	Part A—Prospective observation Part B—Modeling of a supported secondary prevention program	Minor Stroke and Transient Ischemic Attack (aged 18+, with arterial hypertension, diabetes mellitus, atrial fibrillation, and/or smoking	341	• Risk factor control (blood pressure, low density lipoprotein, smoking, physical activity) • Medication adherence	• The support program improved secondary prevention of stroke through improved risk factor control and medication adherence
McNaughton et al. (2021), New Zealand [61]	To examine mechanisms for success of the Take Charge intervention on physical health, advanced activities of daily living, and independence for people post-stroke	Randomized controlled trial	Stroke (living in the community, less than 16 weeks post-stroke)	400	• Physical functioning (Physical Component Summary of the Short Form-36)	• The Take Charge intervention did not significantly promote behaviour change, specific to body mass index, blood pressure control, or medication adherence
Nichols et al. (2019), Ghana [89]	• To explore the experiences of stoke survivors and their caregivers on the mHealth blood pressure management intervention • To explore facilitators and barriers relating to the implementation of the intervention	Qualitative (focus groups)	Stroke	16	• Blood pressure control	• Reflections on the intervention highlighted the participants’ ability to self-monitor blood pressure and adhere to medications, use of technology, and need for ongoing training and support • Medication adherence improved during the intervention, but was not sustained post-intervention
Nir and Weisel-Eichler (2006), Israel [62]	• To evaluate the impact of a multifaceted, tailored nursing intervention on medication use, knowledge, and skills	Interventional study	Stroke (aged 60+)	155	• Correct use of medications • Dietary adherence • Independence in activities of daily living	• Knowledge and skills pertaining to medication therapy (knowledge of medication, side effects, adherence to dietary regimen) increased following the intervention, but was not sustained
O’Carroll et al. (2014), United Kingdom [64]	• To test the mediators and moderators of treatment effects of a brief, personalized intervention	Randomized controlled trial (pilot)	Stroke or Transient Ischemic Attack (discharged home, taking at least one anti-hypertensive medication)	62	• Medication adherence to antihypertensive medication (Medication Events Monitoring System)	• Medication adherence significantly improved following the personalized intervention (through addressing concerns, and developing routines)
O’Carroll et al. (2013), United Kingdom [63]	• To pilot a brief, personalized intervention and assess its impact on medication adherence	Randomized controlled trial (pilot)	Stroke or Transient Ischemic Attack (discharged home, taking at least one anti-hypertensive medication)	62	• Medication adherence to antihypertensive medication (Medication Events Monitoring System)	• Medication adherence significantly improved following the personalized intervention
Olaiya et al. (2017), Australia [65]	• To examine the effectiveness of an individualized management program	Randomized controlled trial	Stroke or Transient Ischemic Attack (aged 18+)	563	• Cardiometabolic factors (blood pressure, lipids, smoking, body weight, blood glucose, kidney function)	• Improved control of lipids at 12 months, no improvements in any other cardiometabolic targets (blood pressure, smoking, body weight, blood glucose, kidney function) at 12 or 24 months
Oura et al. (2021), Japan [66]	• To identify the impact of a medication support device (Pletaal Assist System®) on cilostazol adherence	Pilot and feasibility study	Stroke (aged 20+, treated with cilostazol for 3+ months)	25	• Medication adherence rate (Pletaal Assist System)	• The medication support device may improve adherence to cilostazol in patients with poor adherence
Purdy (2007), Not reported [67]	• To examine the impact of a self-medication program on patient knowledge and independence in medication management	Pre-experimental post-test only comparative group design	Cognitively Impaired Stroke Patients	63	• Logical memory and learning (orientation, thought organization, and reasoning subtests from the Scales of Cognitive Ability in Traumatic Brain Injury	• A greater proportion of patients in the stroke group completed the self-management program and were able to independently manage their medications at discharge
Sajatovic et al. (2018), United States [88]	• To examine the impact of a targeted, self-management intervention on medication adherence, risk factor management, and health behaviours	Prospective randomized controlled trial and qualitative evaluation	Ischemic Stroke or Transient Ischemic Attack (aged 65+, African American men, discharged from hospital within 12 months)	38	• Medication adherence (Tablets Routines Questionnaire)	• Baseline medication adherence rates were high, leaving minimal opportunity for improvement
Sarfo et al. (2019), Ghana [68]	• To assess the effectiveness of an mHealth intervention on improving blood pressure control	Randomized controlled trial	Stroke (aged 18+, stroke within 1 month, uncontrolled hypertension)	60	• Blood pressure	• The mHealth intervention was feasible and demonstrated improvements in blood pressure control
Schwartz et al. (2018), United Kingdom [69]	• To assess the fidelity of a self-management intervention • To determine the accuracy of self-monitored blood pressure reporting	Randomized controlled trial	Stroke (aged 35+, at least one high-risk condition, blood pressure over 130/80 mmHg)	276	• Systolic blood pressure	• The self-management intervention groups had significantly lower blood pressure than the controls • Most self-monitoring was done properly and recorded accurately
Sit et al. (2016), Hong Kong [70]	• To examine the impact of the Health Empowerment Intervention for Stroke Self-management on self-efficacy, self-management behaviour, and functional outcomes	Randomized controlled trial (single blind)	Ischemic and Hemorrhagic Stroke (scheduled for ambulatory stroke rehabilitation, functional difficulties limiting self-care)	210	• Self-efficacy (Chinese Self-Management Behaviour Questionnaire) • Self-management behavior (Chinese Self-Management Behaviour Questionnaire) • Functional outcomes (modified Barthel index and Chinese Lawton instrumental activities of daily living)	• The intervention significantly improved self-efficacy in illness management and self-management behaviours
Sit et al. (2007), Hong Kong [71]	• To identify the effectiveness of a community-based stroke prevention program on stroke knowledge, self health monitoring, and behavioural changes	Quasi-experimental design	Stroke (aged 18+, living in the community, independent in activities of daily living)	190	• Stroke knowledge • Self-health-monitoring practice • Health behaviours (medication adherence, smoking and alcohol consumption, exercise, diet)	• The stroke prevention program significantly improved knowledge of stroke warning signs, treatment-seeking, medication adherence, blood pressure monitoring, and dietary habits
Souter et al. (2017), Not reported [72]	• To assess the practicality, acceptability, and feasibility of a pharmacist complex intervention regarding participant recruitment, data collection, blood pressure monitoring, and pharmaceutical care processes	Randomized controlled trial	Stroke	40	• Feasibility (recruitment, data collection, blood pressure measurement, pharmaceutical care)	• Modifications to recruitment are needed (include transient ischaemic attack) • Questionnaire response rates met criteria but completion rates did not • Reliable processes for blood pressure monitoring are needed
Su et al. (2017), China [73]	• To examine the effects of a health promotion program on adherence to antiplatelet therapy	Controlled multicenter trial	Ischemic Stroke (aged 18+)	1275	• Medication adherence	• The health promotion program improved awareness of secondary prevention and medication adherence
Towfighi et al. (2021), United States [74]	• To identify if risk factor control is improved through a coordinated community and chronic care model team intervention	Randomized controlled trial	Ischemic Stroke, Transient Ischemic Attack, or Intracerebral Hemorrhage (aged 40+, stroke within 90 days, elevated systolic blood pressure	487	• Systolic blood pressure	• No improvements in risk factor control were noted in the intervention or control groups
Vanacker et al. (2017), Belgium [75]	• To examine the feasibility of a personalized stroke coaching program	Retrospective analysis of a prospectively collected cohort	Ischemic Stroke (discharged home)	152	• Clinical outcomes and medication adherence (adapted version of the standardized WSO Post-Stroke Checklist)	• The stroke coaching program was feasible to implement and low rates of stroke recurrence and mortality were noted
Wan et al. (2016), China [76]	• To assess the effectiveness of a goal-setting telephone follow-up program	Randomized controlled trial (single blind)	Stroke (aged 35+, hospitalized within 1 month)	91	• Health behaviour (Chinese version of the Health Promoting Lifestyle Profile II)	• The telephone follow-up program was feasible and resulted in significantly higher medication adherence • No significant differences were identified in physical activity, blood pressure monitoring, low-salt dietary adherence, smoking abstinence, or unhealthy use of alcohol
Wan et al. (2018), China [77]	• To identify if a Comprehensive Reminder System improved health behaviours and blood pressure control	Randomized controlled trial (single blind)	Stroke (hypertension, hospitalized within 1 month, expected discharge home)	174	• Blood pressure • Health behaviour (Chinese version of the Health Promoting Lifestyle Profile II)	• Improvements in blood pressure control and health behaviours (physical activity, low-salt dietary adherence, medication adherence) were identified and were significantly higher in the intervention group
Wang et al. (2021), China [78]	• To evaluate the impact of a pharmaceutical care program on risk factor control and hospital readmissions	Randomized controlled trial	Ischemic Stroke (aged 18+, hypertension and/or diabetes, residing in urban area)	184	• Risk factors • Medication adherence (Medication Adherence Report Scale)	• Significant improvements in medication adherence and risk factor control occurred in the pharmaceutical care group
Wang et al. (2020), China [79]	• To explore the effects on a Comprehensive Reminder System on health behaviours, medication adherence, blood pressure, and degree of disability	Randomized controlled trial (single blind)	Stroke (history or hypertension, stroke within 1 month)	174	• Blood pressure • Blood pressure control rate	• Statistically significant improvements were seen in health behaviours, medication adherence, disability, and blood pressure control
Wang et al. (2020), China [80]	• To explore the effectiveness of a brain and heart health manager-led secondary stroke prevention program on blood pressure, self-management ability, medication adherence, body mass index, and blood low-density lipoprotein	Randomized control trial	Stroke (aged 18+)	200	• Blood pressure • Self-management ability (self-health management scale)	• I mprovements were identified in blood pressure management and self-care ability, but no significant differences in body mass index, total cholesterol, or low-density lipoprotein were noted
Washington-Nash (2017), United States [81]	• To explore the impact of an educational intervention on reading comprehension, health literacy, and self-administration of medications	Quasi-experimental study	Stroke (aged 18–65, female)	28	• Health literacy (Short Test of Functional Health Literacy in Adults) • Reading comprehension (Cloze test)	• Reading comprehension level impacted ability to correctly self-administer medications • Following the intervention, the majority of participants could correctly self-administer medications
Wessol et al. (2019), United States [83]	• To examine the acceptability and feasibility of the SystemCHANGE™ and Attention Control interventions	Pilot randomized controlled trial (single blind)	Ischemic Stroke (aged 50+, prescribed at least 1 daily antithrombotic medication, able to self-administer medication)	2	• Feasibility • Acceptability	• The interventions were identified as feasible and acceptable
Wessol (2018), United States [82]	• To assess the acceptability, feasibility, and effectiveness of the SystemCHANGE™ intervention	Pilot randomized controlled trial (single blind)	Stroke (aged 50+, prescribed at least 1 daily antithrombotic medication, able to self-administer medication)	2	• Feasibility • Acceptability	• The intervention was identified as feasible, without significant participant burden, inconvenience, or time required for participation
Yan et al. (2021), China [84]	• To identify if a primary care-based integrated mobile health intervention improved stroke management	Randomized controlled trial	Stroke	1,299	• Systolic blood pressure	• Significant improvements were identified in blood pressure, hospitalization, mortality, and other health outcomes (medication adherence, disability)
Yan et al. (2020), China [85]	• To determine if a stroke health manager program impacted blood pressure control, low-density lipoprotein cholesterol, glucose level, and use of secondary prevention medications	History-controlled study	Ischemic Stroke (aged 18+)	642	• Systolic and diastolic blood pressure • Blood lipids • Modified Rankin score • Recurrence rate	• The intervention improved blood pressure control, low-density lipoprotein, glucose levels, and persistence to secondary prevention medications
Zhang et al. (2020), China [86]	• To test the feasibility and efficacy of Mobile Phone- and WeChat-Based Improvement Services on secondary stroke prevention and medication adherence	Cohort study	Stroke (aged 18+)	468	• Medication adherence	• Improvements in medication adherence were identified following the self-management program

### Population characteristics

The sample sizes varied, ranging from 2 participants to 5,882 participants (median = 174; IQR = 202). The majority of articles reported the sex (n = 32) [34, 38, 41, 43–49, 52, 53, 56, 57, 59, 61, 65, 66, 68–74, 77–79, 84–86, 88] or gender (n = 19) [35, 37, 39, 40, 42, 51, 54, 55, 58, 60, 62, 63, 75, 76, 80–83, 87] of participants, but no articles reported both sex and gender. Level of education was reported by 29 articles [35, 36, 39, 42, 45, 47, 50–53, 55, 58, 59, 62, 68, 70, 71, 73, 74, 76–81, 84, 87–89], with variability in how education level was collected and how the highest level of education achieved by the participant was reported. Approximately one third of included articles reported the participants’ ethnicity (n = 19) [35, 36, 42–45, 50, 59, 61–64, 69, 74, 81–83, 87, 89], marital status (n = 17) [36, 39, 50, 51, 55, 58, 69–71, 73, 74, 76, 77, 82–84, 88], and employment status (n = 17) [35, 36, 50, 51, 55, 58, 63, 68–71, 76, 82, 83, 87–89]. Income level (n = 12) [45, 46, 51, 52, 55, 58, 59, 62, 68, 73, 76, 81] and household composition (n = 10) [36, 39, 41, 42, 51, 52, 61, 62, 71, 72] were reported less frequently, in about one fifth of included articles. Comorbidities experienced by participants were reported in 27 articles [35, 37, 39, 41, 45, 47, 48, 51, 52, 54, 56–60, 62, 66, 68–70, 73, 76–78, 84, 87, 88], with hypertension, diabetes, and dyslipidemia being the most commonly reported.

### Intervention characteristics

The characteristics of interventions are displayed in Table 2. The goals of the interventions were largely similar across the included studies and focused on: secondary stroke prevention through risk factor management and lifestyle modifications, improving medication-related knowledge, self-efficacy, medication adherence, and quality of life, increasing knowledge of stroke (signs, symptoms, management, risk factors), and improving care coordination and transitions. A component of medication self-management was the primary focus in 13 studies [39, 49, 52, 62–64, 66, 67, 72, 73, 78, 81, 86], with the majority (n = 43) including it as a part of a larger intervention or as an outcome measure. Two-thirds of the interventions consisted of multiple components (n = 42) [34, 35, 38–48, 51–54, 56–60, 62–65, 67–74, 77, 79, 82–85, 87, 88], while the other third were stand-alone interventions (n = 14) [36, 37, 49, 50, 55, 61, 66, 75, 76, 78, 80, 81, 86, 89]. The multicomponent interventions consisted of a combination of the following: education, counseling or coaching, workshops, text reminders, follow-up discussions, information materials, health assessments, referrals or connections to healthcare providers, support services, goal setting, and medication reviews. Just over half of the interventions (n = 33) were tailored to the participants based on their individual needs or goals [34, 37, 38, 40–50, 52–54, 56–59, 62, 63, 65, 67–69, 71, 74, 75, 80, 85, 89]. Only two articles described the modification of the intervention during the course of the study [37, 60]. One study actively modified the length of the sessions depending on the participants’ current state and level of fatigue allowing the sessions to be divided into shorter ones, as needed [37]. The other intervention planned for a possible modification before implementation and modified the intensity of their intervention schemes (module-based support program) [60].

**Table 2:** Intervention characteristics aligning with the TIDieR checklist (n = 56).

Author (year)	What and Why—Intervention Description	Where—Setting	Who—Delivery	When and How much—Frequency	Tailoring	How Well—Results
Annie et al. (2021) [34]	***Secondary stroke prevention*** Educational interventions with booklets to provide information on making lifestyle changes to modifiable risk factors	Outpatient department of a stroke division	Clinical Pharmacist	Not reported	Yes	Significant improvements in medication adherence and disease knowledge post-intervention
Appalasamy et al. (2020) [87]	***Video Narratives*** An intervention delivering video narratives aimed at promoting self-efficacy skills and motivation	Outpatient department of hospital	Researchers	Not reported	Not reported	Improved medication use self-efficacy scores were seen in the intervention and control groups (but greater improvements in intervention) Intervention group showed better blood pressure control
Appalasamy et al. (2020) [35]	***Video Narratives*** An intervention delivering video narratives aimed at promoting medication use self-efficacy	Outpatient department of hospital	Neurologists and researchers	Not reported	Not reported	Significant improvements in medication use self-efficacy scores for antithrombotic, antihypertensive, and all medication categories Trends for improved illness perceptions, knowledge, and beliefs about medicines
Barker-Collo et al. (2015) [36]	***Motivational Interviewing*** Promotes behaviour change through structured, patient-centred motivation and aims to increase medication adherence and knowledge around potential problems, risks, and effects of certain behaviours	Hospital and primary residence	Trained researchers	Four sessions at 1, 3, 6, and 9 months post-stroke	Not reported	No significant changes in blood pressure or cholesterol Improved self-reported medication adherence
Ben Nasr et al. (2018) [37]	***Educational Program*** A program targeting secondary stroke prevention through education on disease management, medication adherence, blood pressure management, and health practices	Stroke centre	Multi-professional team (physicians, nurses, pharmacist)	Mean duration of 46 minutes	Yes	Self-reported medication adherence, frequency of blood pressure measurements, and patient knowledge improved post-educational program Participants were satisfied with the program
Bretz et al. (2014) [38]	***National Stroke Association Steps Against Recurrent Stroke (STARS) Plus Program*** A program designed to deliver preventative strategies and promote improved quality of life	Stroke centres	Not reported	Not reported	Yes	Increased medication adherence, high patient satisfaction, and reduced rehospitalization Increased subjective pain
Chandrasekhar et al. (2018) [39]	***Pharmaceutical Care*** An interaction between the patient and pharmacist aimed at improving adherence and quality through counseling and follow-ups	Neurology department in a private tertiary care referral hospital	Pharmacist	Not reported	Not reported	Significant improvements in medication adherence and health-related quality of life post-intervention
Chen et al. (2016) [40]	***Home Care Model*** A model of care that promotes care coordination through continuous nursing services and rehabilitation provided by specialized medical staff, or home and community nurses	Comprehensive teaching hospital	Team of chief physician, clinical pharmacist, psychologist, specialist dietitian, rehabilitation therapist, specialist, and community nurse	Home visit during the first week post-discharge, weekly home visits for 5 weeks, weekly telephone calls	Yes	Significantly better outcomes in the intervention group: shorter average length of hospitalization, higher medication compliance, and greater satisfaction, ability to perform activities of daily living and satisfaction
Coombes et al. (2020) [41]	***Patient‐Centred Educational Exchange (PC3EE)*** An intervention consisting of education sessions targeting stroke knowledge, medications, and medication-taking behaviour	Stroke unit of tertiary teaching hospital	Educator (clinical pharmacist)	Two sessions	Yes	The use of the questionnaires to assess knowledge and beliefs was feasible and acceptable
Damush et al. (2016) [42]	***Patient-based Veterans Stroke Prevention Program*** A program focused on improving patient self-efficacy in managing post-stroke symptoms	Veterans Administration Medical Centres	Nurse case managers	Two calls per week	Yes	No significant improvements in self-efficacy, or health-related quality of life between intervention and control groups Improved medication adherence within intervention group
Deen et al. (2018) [43]	***Stroke Nurse Navigation Program (SNNP)*** A program designed to improve transitions from acute care to home by identifying and addressing the needs of patients and caregivers	Community hospital and primary stroke centre	Nurse navigators	Five follow-up phone calls at 7 and 30 days, 3, 6, and 12 months	Yes	Trends towards improved medication adherence, activities of daily living, quality of life, and follow-up appointments, reduced rehospitalization
Duncan et al. (2020) [44]	***Comprehensive Post Acute Stroke Services (COMPASS)*** A complex intervention using individualized care plan consisting of education, secondary prevention, recovery, referrals to community services, and caregiver supports	Hospital and community	Post-acute care nurse coordinator, advanced practice provider or physician	Telephone follow-up within two days of discharge, clinic visit 7–14 days post-discharge, 2-day training	Yes	Functional status did not significantly improve post-intervention Blood pressure monitoring was significantly more prevalent in the intervention group Survival, satisfaction with care, depression, disability, and falls were improved in the intervention group
Evans-Hudnall et al. (2014) [45]	***Cognitive behavioral therapy-based secondary stroke prevention (STOP) treatment*** Three self-care, cognitive behavioural sessions that included information on self-monitoring, problem-solving, cognitive restructuring, stress management, stimulus control, goal setting, social support, and relapse prevention	Hospital and community	Health educator	Three 30–45 minute sessions	Yes	Significant differences between the intervention and control groups for stroke knowledge and tobacco use
Feng et al. (2021) [46]	***Hospital-community integrated service model (HCISM)*** An integrated model of care targeting post-discharge needs–reducing family burden, enhancing service capabilities of staff, and improving quality of life	Hospital and community	Team of neurologists, rehabilitation therapists, nurses, community doctors	Telephone follow-up, once per month post-discharge, telephone follow-up twice per month in community, WeChat as needed, home visits one per month	Yes	Activities of daily living improved in the intervention group compared to control Diet, exercise, follow-up visits, negative emotions, self-efficacy, and medical compliance behaviour improved in the intervention group
Fruhwirth et al. (2022) [47]	***PRESTRO*: *Prevent Stroke App Intervention*** A smartphone app designed to prevent secondary stroke through motivation, reminders for medication and blood pressure measurements, and stroke education	Dedicated outpatient stroke department	Psychologists	Not reported	Yes	Individuals in the intervention group were significantly more active than the control group and had better health-related quality of life Smoking behaviour and hypertension decreased in all patients
Hedegaard et al. (2014) [48]	***Clinical Pharmacist Intervention with Motivational Interviewing*** An intervention using a counseling approach to promote behaviour change and improve empowerment	Hospital and community	Clinical pharmacists	Telephone calls at 1 week, 2 and 6 months post-discharge	Yes	Medication adherence and persistency to secondary stroke therapy did not significantly improve post-intervention There was no significant impact on clinical outcomes
Hohmann et al. (2013) [49]	***Medication Information*** A detailed discharge letter containing medication-related information (medication at admission and discharge, medication changes, reasons for new medications, discontinued medications, modifications	Hospital	Clinical pharmacist	Not reported	Yes	Significantly better adherence to antithrombotic drugs and statin therapy in the intervention group compared to control
Holzemer et al. (2011) [50]	***Educational Intervention*** Individualized education plans delivered post-stroke to promote lifestyle changes and risk factor control	Hospital	Hospital staff nurses, stroke team	Not reported	Yes	Adherence to risk-factor modification improved significantly
Kalav et al. (2022) [51]	***StrokeCARE Intervention*** An intervention based on the Chronic Care Model designed to improve self-efficacy, quality of life, activities of daily living, and metabolic control	Neurology clinic of hospital	Health team	Telephone follow-up post-discharge at 7 and 14 days, and 4 and 8 weeks, reminders at 1^st^, 2^nd^, 3^rd^, 4^th^, 6^th^, 8^th^, and 10^th^ weeks	Not reported	No significant differences in the intervention and control groups for self-efficacy or quality of life Significantly higher satisfaction scores in the intervention group
Kamal et al. (2018) [52]	***Talking Prescriptions (Rx)*** A tailored health technology intervention designed to improve medication adherence in environments with limited resources	Hospital	Research and clinical teams	Daily	Yes	No significant improvements in medication adherence The intervention was feasible
Kamal et al. (2015) [53]	***Short Messaging Service (SMS)-SMS4Stroke*** A mobile technology intervention designed to promote behaviour change and improve medication adherence	Clinical Trials Unit at hospital	Research and stroke teams	Daily	Yes	Significant improvements in medication adherence and blood pressure The intervention was feasible and acceptable and participants were highly satisfied
Kamoen et al. (2020) [54]	***Stroke Coach and Digital Platform*** A secondary stroke prevention program that consists of patient participation, coaching, and telemedicine	Hospitals with a dedicated stroke unit	Stroke coach (specialized nurse under supervision of stroke neurologist)	One session during hospitalization, video consultations at 2 weeks, 1, 2, and 6 months post-discharge, face-to-face follow-up at 3 months	Yes	Significant reduction in the intervention group in mean Systematic COronary Risk Evaluation (SCORE), but insignificant difference between intervention and control groups High self-reported medication adherence and quality of life
Kim and Park (2011) [55]	***Web-based Education Program*** An educational intervention addressing knowledge of secondary stroke prevention, family support, health behaviours, medication adherence, smoking cessation, and health status	Outpatient clinic of hospital	Discharge training provided by the hospital	Discharge training, six telephone calls every two weeks beginning two weeks post-discharge	Not reported	Significantly higher knowledge of secondary stroke prevention, health behaviour adherence, and family support in intervention group compared to control No significant differences in medication adherence, blood pressure, or perceived health status
Kim et al. (2020) [56]	***Mobile Health Care System*: *Smart Aftercare*** A multifaceted, mHealth platform consisting of wearable devices, a post-stroke app, and clinician monitoring in order to improve stroke awareness, mood, and quality of life	Medical centre	Clinicians (professions not specified)	Three visits over 12 weeks	Yes	Insignificant improvements in stroke awareness, mood, and blood pressure Improvements in medication adherence
Kitzman et al. (2017) [57]	***Kentucky Care Coordination for Community Transitions (KC3T)*** A program designed to improve the transition to rural communities post-stroke by facilitating communication and setting up services/ resources	Inpatient rehabilitation hospital	Community health workers	At least once per week for the first three months and once every other week for months 4–6	Yes	92% of participants adhered to their medications 96% attended all scheduled physician visits; 70% attended all scheduled outpatient rehabilitation visits
Komton (2021) [58]	***SystemCHANGE***™—***Stroke*** A behaviour change intervention designed to modify routines and improve healthy eating, physical activity, and medication adherence	Stroke units at hospital	Trained interventionists (registered nurses and a nursing faculty member)	Initial home visit within 2 weeks, four face-to-face group sessions, three calls every 4 weeks	Yes	The intervention improved medication adherence, healthy eating, and physical activity
Kronish et al. (2014) [59]	***Peer Education*** A peer-led, educational intervention consisting of weekly workshops addressing self-management skills	Inner city, New York City	Peer leaders with similar socio-economic backgrounds and health problems as participants	Six weekly peer-led workshops	Yes	No significant differences between the intervention and control groups for low density lipoprotein cholesterol or antithrombotic use Greater blood pressure control in the intervention group compared to control
Leistner et al. (2012) [60]	***Module-based Support Program*** A support program consisting of five educational and behaviour modules–blood pressure, physical activity, anticoagulant/ antiplatelet therapy, nicotine cessation, and nutrition and metabolism counseling	Stroke unit department at hospital	Doctors and prevention assistants	Three to four appointments	Yes	The support group had significantly better risk factor control, medical compliance, and blood pressure
McNaughton et al. (2021) [61]	***Take Charge*** An intervention designed to facilitate self-management rehabilitation post-stroke	Community	Research clinicians (nurses, physiotherapists)	One session or two sessions lasting 30–60 minutes	Not reported	No significant differences in activation, mood, medication adherence, body mass index, ability to Take Charge, or blood pressure
Nichols et al. (2019) [89]	***Phone-based Intervention under Nurse Guidance after Stroke (PINGS)*** A smartphone, mHealth application for monitoring and improving blood pressure control and medication adherence with the delivery of tailored and motivational text messages	Not reported	Not reported	Daily	Yes	Participants were receptive to home blood pressure monitoring and the use of the mHealth app Access to PINGS facilitated improved adherence, but this was not maintained post-intervention
Nir and Weisel-Eichler (2006) [62]	***Nursing Intervention*** A tailored nursing intervention following a structured guidebook to improve medication knowledge and skills post-stroke	Hospital and community	Student nurses	One session per week for 1–2 hours (for 12 weeks)	Yes	The intervention group had significantly better medication knowledge about the colour, shape, dosage, and number of times per day Improved knowledge of side effects and response to side effects
O’Carroll et al. (2014) [64]	***Brief Intervention*** A personalized intervention consisting of educational sessions designed to improve adherence to preventative medication	Research facility at hospital or community (participant’s home)	Research fellow (health psychologist)	Two sessions, two weeks apart	Not reported	Reduced concerns about medications Significantly greater medication adherence in the intervention group
O’Carroll et al. (2013) [63]	***Brief Intervention*** A personalized intervention consisting of educational sessions designed to improve adherence to preventative medication	Research facility at hospital or community (participant’s home)	Research fellow (health psychologist)	Two sessions, two weeks apart	Yes	Reduced concerns about medications Significantly greater medication adherence in the intervention group
Olaiya et al. (2017) [65]	***Shared Team Approach between Nurses and Doctors for Improved Risk factor Management*** Secondary stroke prevention consisting of a management program and educational sessions to improve cardiometabolic risk factors	Not reported	Nurse	Three education sessions	Yes	No differences between the intervention and control groups for achieving cardiometabolic targets Poor uptake of lifestyle and behavioural habits
Oura et al. (2021) [66]	***Pletaal Assist System*** A medication support device that aims to improve adherence through the recording of medication information	Not reported	Pharmacist	Daily	Not reported	No significant differences in medication adherence rates before, during, or after the Pletaal Assist System Patients with an adherence rate of less than 100% improved during the intervention
Purdy (2007) [67]	***Self-Medication Program*** A program designed to improve knowledge and independence with medication management	Not reported	Primary nurse and pharmacist	Not reported	Yes	81% of stroke patients were able to independently manage their medications at discharge
Sajatovic et al. (2018) [88]	***TargetEd MAnageMent Intervention (TEAM)*** An intervention consisting of individual and group educational sessions to improve self-management post-stroke	Not reported	Nurse educator	12 sessions over 6 months	Not reported	Significantly lower mean blood pressure in intervention group Improved awareness of risk factors
Sarfo et al. (2019) [68]	***Phone-based Intervention under Nurse Guidance after Stroke (PINGS)*** A smartphone, mHealth application for monitoring and improving blood pressure control with the delivery of tailored and motivational medication adherence text messages	Outpatient neurology clinic at a teaching hospital	Nurse	Monthly	Yes	Confidence in taking medications as prescribed and self-autonomous regulation scores improved significantly
Schwartz et al. (2018) [69]	***Targets and Self-Management for the Control of Blood Pressure in Stroke and at Risk Groups (TASMIN-SR)*** A self-management intervention in which patients self-monitored their blood pressure and made medications changes with their provider	Not reported	Not reported	Two sessions with a third if required	Yes	The self-management intervention was feasible; the protocol was followed and self-monitoring was accurately reported 70% of algorithm-recommended medication changes were made
Sit et al. (2016) [70]	***Health Empowerment Intervention for Stroke Self-management (HEISS)*** A health empowerment intervention designed to enhance self-efficacy, self-management, and functional recovery post-stroke through group sessions and follow-up telephone calls	Ambulatory rehabilitation centre of a subacute hospital	Nurse	Part 1: Six weekly small group sessions Part 2: Biweekly phone calls over 5 weeks	Not reported	The intervention group had significantly better self-efficacy, self-management behaviours, and functional recovery
Sit et al. (2007) [71]	***Community-Based Stroke*** ***Prevention Program*** A secondary stroke prevention program designed to improve stroke knowledge, self-monitoring, healthy habits through group sessions targeting individualized goal setting and action plans	Not reported	Community nurses	Eight 2-hour sessions, once per week	Yes	Significant effect on knowledge of stroke warning signs, treatment seeking response, medication adherence, blood pressure monitoring, and dietary habits in intervention group No significant improvement in participation in walking exercise
Souter et al. (2017) [72]	***Pharmaceutical Care*** A complex intervention consisting of a pharmaceutical care plan, individualized patient information, medication review, and medication and lifestyle advice	Community (participant’s home)	Clinical pharmacist	Three home visits and information sheet	Not reported	Blood pressure and lipid measurement processes were not reliable Questionnaire response rates met criteria, but completes rates did not, further feasibility testing is needed
Su et al. (2017) [73]	***Health Promotion Program*** A program designed to improve medication adherence to antiplatelet therapy through the delivery of a secondary prevention manual, training sessions, and follow-up telephone calls	Hospital and community	Physicians (trained as health coaches)	30 minutes per day for three days, telephone calls at 1, 3, and 6 months	Not reported	Significant increase in the proportion of patients on antiplatelet therapy, as well as awareness of antiplatelet therapy in the intervention group
Towfighi et al. (2021) [74]	***Secondary Stroke Prevention by Uniting Community and Chronic Care Model Teams Early to End Disparities (SUCCEED)*** An intervention to improve secondary stroke prevention and care coordination through workshops, telephone visits, tailored education, life and risk factor management, medication adherence, and self-management skills	Clinic and community (home visits)	Team of advanced practice clinician, community health worker, physician	At least three clinic visits and three home visits, and telephone calls	Yes	Insignificant improvements in blood pressure Improvements in salt intake in the intervention group compared to control
Vanacker et al. (2017) [75]	***Individualized Coaching Program*** A personalized coaching program delivered post-stroke to assess problems and needs, and to discuss risk factor management, therapy, and clinical evolution	Hospital and community	Stroke nurse	Twice during hospitalization, post-discharge calls at 2 weeks, 1, 3, 4, and 12 months as needed	Not reported	The personalized coaching service was feasible Reduced stroke recurrence and mortality
Wan et al. (2016) [76]	***Goal-Setting Telephone Follow-Up Program*** A telephone-based intervention focusing on pre-discharge education and goal-setting sessions to improve secondary stroke prevention	Neurology departments in hospital and community	Stroke nurses	Three follow-up calls after discharge (at 1 week, 1 month, 3 months)	Not reported	Significantly better medication adherence in the intervention group No significant differences in physical activity, nutrition, low-salt diet adherence, blood pressure monitoring, smoking abstinence, or unhealthy alcohol use
Wan et al. (2018) [77]	***Comprehensive Reminder System based on the Health Belief Model (CRS-HBM)*** A program focused on improving patients’ health behaviours through transitional care, health education, a calendar handbook, automated short messages, and telephone follow-ups	Neurology departments in hospital	Research nurses	Not reported	Not reported	Significantly improved health behaviours for physical activity, nutrition, low-salt diet, medication adherence, blood pressure, and blood pressure control in the intervention group compared to control
Wang et al. (2021) [78]	***Pharmaceutical Care*** An intervention consisting of in-hospital care and post-discharge follow-up education on medication therapy management, disease management, and secondary prevention of stroke	Hospital	Clinical pharmacist	Seven times over six months	Not reported	Significant improvement in medication adherence in intervention group More patients in the intervention group achieved risk factor control markers for hemoglobin and low density lipoprotein
Wang et al. (2020) [79]	***Comprehensive Reminder System based on the Health Belief Model (CRS-HBM)*** An intervention consisting of health education, a calendar handbook, automated short messages, and telephone follow-ups to improve health behaviours and blood pressure control	Community (participant’s home)	Nurses	Not reported	Not reported	Improved (insignificant) health behaviours, medication adherence, blood pressure, and disability in the intervention group compared to control
Wang et al. (2020) [80]	***Brain and Heart Health*** ***Manager-led (BHHM) mHealth Secondary Stroke Prevention*** A secondary stroke prevention program consisting of a monitoring service, health service, hospital service, and doctor service	Community (participant’s home)	Brain and heart health manager	Daily	Yes	Significant difference in medication adherence and self-care ability between intervention and control groups Improvements were not identified at one year for body mass index, total cholesterol, or low density lipoprotein
Washington-Nash (2017) [81]	***Nurse-led Educational Intervention*** An intervention consisting of a handout and instruction on the self-administration of medications aimed at improving health literacy	Health care settings	Nurse	Not reported	Not reported	Majority of participants could correctly self-administer medications post-educational intervention
Wessol et al. (2019) [83]	***Change Habits by Applying New Goals and Experiences (SystemCHANGE™)*** A behaviour change intervention to help redesign daily routines to improve medication adherence and healthy living	Community (participant’s home)	Principal investigator (registered nurse)	Daily	Not reported	The intervention was feasible and acceptable, but more research is needed to determine the effect on medication adherence
Wessol (2018) [82]	***Change Habits by Applying New Goals and Experiences (SystemCHANGE™)*** A behaviour change intervention to help redesign daily routines to improve medication adherence and healthy living	Community (participant’s home)	Principal investigator (registered nurse)	Daily	Not reported	The intervention was feasible, acceptable, positive, and not a burden to participants
Yan et al. (2021) [84]	***Primary Care-Based Integrated Mobile Health Intervention (SINEMA)*** A mobile app designed to support blood pressure control and secondary stroke prevention through follow-up visits, reminders, training/ education, and performance indicators	Community	Doctor	Monthly, with messages sent daily	Not reported	The program had high fidelity Significant improvements in blood pressure, health-related quality of life, level of physical activity, medication adherence, as well as reduced stroke recurrence, disability, and mortality in intervention group
Yan et al. (2020) [85]	***Stroke Health Manager*** An educational program for patients and families post-stoke to improve health status, risk factor control, and quality of life Consisted of health education, discharge advice, access to WeChat, and clinical consultations	In-patient department of hospital and community	Stroke health manager	Every workday afternoon	Yes	Significantly improved blood pressure, low density lipoprotein cholesterol, glucose control, and medication persistence in intervention group
Zhang et al. (2020) [86]	***WeChat-Based Improvement Services and Self-Monitoring Platform*** A communication app that integrates self-monitoring, reminders, and medication-related modules to improve treatment adherence	Community healthcare service centre	N/A—App	Not reported	Not reported	Medication adherence improved in the WeChat monitoring group

The majority of interventions were delivered by nurses (n = 15) [42, 43, 54, 61, 62, 65, 68, 70, 71, 75–77, 79, 81, 88], a multidisciplinary clinical team (n = 11) [35, 37, 40, 46, 50–53, 60, 67, 74], or pharmacists (n = 7) [34, 39, 48, 49, 66, 72, 78] and were initiated in a hospital or healthcare setting (n = 40) [34–60, 62, 68, 70, 73–77, 80, 81, 85–87], with 11 of those including a cross-sectoral or community-based component [36, 44–46, 48, 62, 73–76, 85]. Fewer interventions were delivered to individuals in the community only (n = 9). The setting was not clearly reported in 7 articles [65–67, 69, 71, 88, 89]. Seventeen interventions used only in-person methods for delivery [34, 37, 39, 49, 59–65, 67, 69, 71, 72, 81, 88] and six used only technological mechanisms for delivery [47, 52, 53, 66, 86, 89]. However, most interventions leveraged the use of both face-to-face interactions and technology for delivery (n = 33) [35, 36, 38, 40–46, 48, 50, 51, 54–58, 68, 70, 73–80, 82–85, 87]. The technological component of the interventions consisted of telephone follow-ups, coaching, interviews or hotlines, informational or motivational videos, online chats, text reminders, education, and mobile applications (e.g., mHealth apps). Most interventions were conducted on an individual basis (n = 47) [34–41, 43–45, 47–56, 60–69, 72–84, 86, 87, 89], rather than in a group setting (n = 2) [59, 71]. Seven interventions included both individual and group components [42, 46, 57, 58, 70, 85, 88]. There was significant variation in the frequency and duration of the interventions. The frequency ranged from daily to a total of two sessions and the duration ranged from 10 days to 12 months.

### Intervention outcomes

Outcomes specific to each of the interventions are displayed in Table 2. The included interventions evaluated a number of different outcomes, which were categorized as behavioural outcomes [32], learning outcomes [31], and clinical outcomes [33]. Behavioural outcomes (e.g., medication adherence, compliance, or persistence, physical activity, blood pressure monitoring, nutrition) were the most commonly targeted outcomes across the interventions. Medication adherence was assessed as a primary or secondary outcome in 34 interventions. Of these 34 interventions, the majority improved medication adherence. However, no positive impacts on medication adherence were found in four studies [48, 52, 61, 66]. Learning outcomes included self-efficacy and knowledge. Knowledge was evaluated in eight interventions and demonstrated improvements related to knowledge about stroke, secondary prevention, medications, side effects, and response to side effects [34, 35, 37, 41, 45, 55, 62, 71]. Self-efficacy was assessed in six interventions [35, 42, 46, 51, 70, 87], with two-thirds (n = 4) showing improvements [35, 46, 70, 87]. The concept of self-management was evaluated using a specific self-management outcome measure for tasks, skills, or behaviours in four studies [37, 51, 70, 80], demonstrating favourable outcomes in three [37, 70, 80]. The clinical outcomes (e.g., blood pressure, low-density lipoprotein, depression/ mood, quality of life) evaluated across the included studies did not directly align with medication self-management, but may have been impacted by self-management tasks, skills, or behaviours.

### Discussion

The purpose of this scoping review was to identify what was reported in the literature on interventions for medication self-management for adults who experienced a stroke. Based on the 56 included articles, we found that: (1) there were fewer interventions (n = 13) that specifically targeted a component of medication self-management; (2) the majority of interventions were initiated in-hospital or in a healthcare setting, rather than in the participants’ own community environment; (3) there is limited reporting of the ideal frequency, duration, and sustainability of the interventions to improve outcomes; and (4) interventions frequently focused on quantitative outcome measures, with limited qualitative exploration of implementation considerations and experiences.

We found that the majority of interventions did not specifically target medication self-management, but rather incorporated a small component, such as medication adherence, as part of a broader intervention focused on changing health behaviour. Importantly, medication self-management encompasses the tasks, skills, and behaviours related to one’s navigation of the physical, social, and cognitive lifestyle factors, changes, and consequences inherent in taking, or not taking, medications. Despite medication self-management including all these components, the majority of literature to date and available supports focus heavily on medication adherence. Given the wide range of challenges individuals who experienced a stroke face related to medications (e.g., medication burden, medication understanding, medication-taking self-efficacy, medication management [17–20]), it is important for interventions to focus on these areas in order to comprehensively address medication self-management. Our review identified only one study that took a more focused approach at medication self-management [62]. Specifically, this study involved a tailored and multifaceted nursing intervention that followed a structured guidebook to improve individual’s knowledge and skills with medication use and dietary habits post-stroke [62]. The intervention addressed affective (e.g., trusting environment, coping, self-esteem, self-control, self-empowerment, decision-making, self-confidence), instrumental (self-care skills, accepting health status, increasing medication self-management capability), and cognitive aspects of medication self-management (perceptions, attitudes, and beliefs of illness, self-management of medications, memory techniques, medication understanding, skills, follow-up visits and clinical tests). Significant positive effects were seen in the intervention group compared to controls for knowledge of medication shape and dosage, side effects, and response to side effects, but they were not sustained at six months. This was because the control group had increased their knowledge in these areas, eliminating the significant difference between the groups. The authors of that study suggested that the participants in the control group may have learned on their own or received education from other healthcare providers in the community. The authors also suggested that the intervention may have been more beneficial if it was delivered several months post-stroke once patients’ medication regimens were stabilized and if additional time was spent on medication self-management.

Most interventions were initiated in-hospital or in a healthcare setting (n = 40), with few initiated in the community (n = 9). Unfortunately, while in hospital, patients are often not actively involved in the management of their medication regimens and do not have a full understanding of what is involved until they are discharged and confronted with day-to-day challenges (e.g., cost, establishing routines, physically taking medications, concerns about medication use) [90, 91]. Following a stroke, the return to the individuals’ pre-hospital residence is often the goal [92]. There is evidence reported by previous research exploring discharge locations post-stroke, where home is the most common location, with rates of patients returning home ranging between 63% and 83% [93–95]. Based on the large percentage of individuals who are returning home post-stroke [93–95], it is important to have access to services and programs in the community to support medication self-management. This need was also noted by Gibson and colleagues who conducted a qualitative study with persons who experienced a stroke, caregivers, and nurses and highlighted the importance of having strategies to improve medication adherence once discharged from hospital [91]. General chronic disease self-management programs delivered in the community have shown promising outcomes for persons who experienced a stroke with improving self-efficacy, health behaviours, and quality of life [96], and can offer key learnings to apply to medication self-management programs. Furthermore, the return home for the majority of patients post-stroke emphasizes the importance of having medication self-management programs available in the community, as individuals and their caregivers learn to navigate a new norm and are responsible for most, if not all, aspects of their medication regimen.

In this scoping review we identified immense variation in the frequency (daily to total of two sessions) and duration (10 days to 12 months) of interventions related to medication self-management for those with stroke. While the variation may be attributed to a number of different factors (e.g., country, health system, funding), there is a need to better understand the optimal frequency and duration of interventions to maximize patient outcomes and experiences, while also maintaining sustainability over time. The Integrated Sustainability Framework by Shelton and colleagues presents multilevel factors (outer contextual factors, inner contextual factors, processes, intervention characteristics, and implementer characteristics) that facilitate the sustainability of interventions across settings and in different contexts [97]. These factors should be considered during the development, implementation, and adaptation of interventions, rather than once implementation has occurred. As such, when developing interventions, Shelton and colleagues recommend the use of sustainability theory as part of the planning process [97]. Overall, this scoping review identified a lack of consistency in the frequency and duration of the interventions, with limited understanding of their sustainability over time. The sustainability of interventions is critical to continually improve patient outcomes and experiences, and thus, should be considered as part of the design process.

Most studies were quantitative, with few using mixed methods or qualitative study designs. Qualitative research can supplement and provide important context to quantitative outcome measures by answering the ‘how’s’ and ‘why’s’ during development, implementation, and evaluation of interventions [98, 99]. For example, qualitative research can contribute to a better understanding of the feasibility, acceptability, and appropriateness of the intervention by allowing for a more in-depth discussion of these outcomes by those who were involved (as participants or as implementers). Understanding how participants and individuals implementing the intervention perceived it (frequency, duration, outcomes, delivery, overall experiences) can also support sustainability by identifying challenges and adapting those areas. Further to this, qualitative methods can allow researchers to explore, not only if an intervention was successful in achieving quantitative targets, but how it was successful, why it was successful, who it worked for, in what setting, and when [99]. Given the infancy of published mixed methods and qualitative research in this area, there is an opportunity to expand the collective knowledge around how individuals with stroke experience the interventions in which they participate, as well as why interventions work, for whom, when, and where.

### Gaps and opportunities for future research

Based on the findings from this scoping review, key areas requiring future research include the following: (1) interventions that comprehensively address medication self-management beyond adherence; (2) interventions that are delivered across sectors or in the community in order to better address self-management, including the assessment of outcomes depending on location of delivery; (3) mixed methods studies to develop a better understanding of what frequency and duration of intervention delivery is most feasible but that will also yield the maximum benefit (through sustainability); and (4) qualitative studies to explore how individuals experience the interventions, including feedback for ongoing adaptation and improvement. While extending research in these areas, it is also important to collect and report on the cognitive level of the participants to better understand who benefits, or does not, from specific interventions.

## Limitations

There are a few limitations of this scoping review to note. First, despite a comprehensive search of five electronic databases and grey literature, it is possible that relevant articles were missed because we excluded conference abstracts, opinion pieces, protocols, and articles in which we could not access the full-text. The University of Toronto is the largest academic library in Canada and has an extensive catalogue of resources [100], but there were instances where we could not access full texts from this system or from the interlibrary loan system. Second, self-management is not a well or consistently defined term. While we tried to be comprehensive in our search for self-management and self-management related tasks, skills, and behaviours, it is possible that articles were missed due to the terms searched. Similarly, in this review, we did not explore medication self-management support (from caregivers, healthcare providers, etc.) for individuals who have experienced a stroke, which is a key area of future work. Third, we did not get a professional translation of the articles published in a language other than English (n = 1), so it is possible that some details were missed or not entirely accurate during data extraction. Lastly, while not a requirement of scoping reviews, we did not conduct a critical appraisal of included articles [27].

## Conclusions

This scoping review included 56 articles related to medication self-management for adults with stroke. While there were several studies that incorporated medication adherence into a larger intervention, there were few that specifically targeted medication self-management. There remains an opportunity to better support medication self-management for adults with stroke by comprehensively addressing all areas of self-management, delivering interventions across sectors or in the community to ensure individuals are in an environment where they self-manage, understanding the optimal frequency and duration of interventions while maintaining sustainability, and qualitatively exploring experiences with interventions.

## Supporting information

S1 Table Preferred Reporting Items for Systematic reviews and Meta-Analyses extension for Scoping Reviews (PRISMA-ScR) checklist. (DOCX)

S2 Table Full search strategies for all electronic databases. (DOCX)

S3 Table List of full-text reports excluded by reason (n = 106). (DOCX)
