# Supplementary material for: Medication self-management interventions for persons with stroke: A scoping review
Source: PLoS One. 2023 May 18;18(5):e0285483. doi: 10.1371/journal.pone.0285483 (PMC10194931; doi:10.1371/journal.pone.0285483)
Supplement: S3 Table — (DOCX) [file pone.0285483.s003.docx]

**S3. List of Full-Text Reports Excluded by Reason (n=106)**

| **Title** | **Authors** |
| --- | --- |
| ***Not at least 50% adults with SCI/stroke (n=12)*** | |
| Development of an aphasia-friendly warfarin consultation tool for patients after stroke | Bhandal, Satinder; Barnett, Nina; Clarkson, Katrina |
| Impact of a Culturally Tailored mHealth Medication Regimen Self-Management Program upon Blood Pressure among Hypertensive Hispanic Adults | Chandler, J.; Sox, L.; Kellam, K.; Feder, L.; Nemeth, L.; Treiber, F. |
| Opportunities for Technology: Translating an Efficacious Intervention to Improve Medication Adherence Among Older Adults | Insel, K.; Lee, J. K.; Einstein, G. O.; Morrow, D. G. |
| A scoping review of polypharmacy interventions in patients with stroke, heart disease and diabetes | Lum, M. V.; Cheung, M. Y. S.; Harris, D. R.; Sakakibara, B. M. |
| Effect of Self-monitoring and Medication Self-titration on Systolic Blood Pressure in Hypertensive Patients at High Risk of Cardiovascular Disease The TASMIN-SR Randomized Clinical Trial | McManus, R. J.; Mant, J.; Haque, M. S.; Bray, E. P.; Bryan, S.; Greenfield, S. M.; Jones, M. I.; Jowett, S.; Little, P.; Penaloza, C.; Schwartz, C.; Shackleford, H.; Shovelton, C.; Varghese, J.; Williams, B.; Hobbs, F. D. R. |
| Characteristics of self-care interventions for patients with a chronic condition: A scoping review | Riegel, B.; Westland, H.; Iovino, P.; Barelds, I.; Slot, J. B.; Stawnychy, M. A.; Osokpo, O.; Tarbi, E.; Trappenburg, J. C. A.; Vellone, E.; Stromberg, A.; Jaarsma, T. |
| A long-term-care setting pilot study evaluating predictors of success in medication self-administration | Schepers, G. P.; Won, H. K.; Bieliauskas, L. A.; Galecki, A. T.; Hogikyan, R. V. |
| A Collaboration Among Primary Care-Based Clinical Pharmacists and Community-Based Health Coaches | Sorensen, A.; Grotts, J. F.; Tseng, C. H.; Moreno, G.; Maranon, R.; Whitmire, N.; Viramontes, O.; Atkins, S.; Sefilyan, E.; Simmons, J. W.; Mangione, C. M. |
| A Cluster-Randomized, Controlled Trial of a Simplified Multifaceted Management Program for Individuals at High Cardiovascular Risk (SimCard Trial) in Rural Tibet, China, and Haryana, India | Tian, M. Y.; Ajay, V. S.; Dunzhu, D. Z.; Hameed, S. S.; Li, X.; Liu, Z.; Li, C.; Chen, H.; Cho, K. W.; Li, R. L.; Zhao, X. S.; Jindal, D.; Rawal, I.; Ali, M. K.; Peterson, E. D.; Ji, J. C.; Amarchand, R.; Krishnan, A.; Tandon, N.; Xu, L. Q.; Wu, Y. F.; Prabhakaran, D.; Yan, L. J. L. |
| The effect of health education on self-care behaviors and hypertension control in elderly hypertensive patients at a veterans home | Tu, M. S. |
| Reducing everyday memory and planning problems by means of a paging system: a randomised control crossover study | Wilson, B. A.; Emslie, H. C.; Quirk, K.; Evans, J. J. |
| The effect of nurse home-support programme on self-management of the patients receiving oral anticoagulation (Warfarin) therapy | Yildirim, J. G.; Temel, A. B. |
| ***No intervention aimed at medication management (n=9)*** | |
| e-Health solution for home patient telemonitoring in early post-acute TIA/ Minor stroke during COVID-19 pandemic | Ajcevic, M.; Furlanis, G.; Naccarato, M.; Caruso, P.; Polverino, P.; Marsich, A.; Accardo, A.; Manganotti, P. |
| Medication adherence: WHO cares? | Brown, M. T.; Bussell, J. K.; Brown, Marie T.; Bussell, Jennifer K. |
| Self-management of pain and depression in adults with spinal cord injury: A scoping review | Cadel, L.; DeLuca, C.; Hitzig, S. L.; Packer, T. L.; Lofters, A. K.; Patel, T.; Guilcher, S. J. T. |
| Predicting compliance with treatment following stroke: A new model of adherence following rehabilitation | Coetzee, N.; Andrewes, D.; Khan, F.; Hale, T.; Jenkins, L.; Lincoln, N.; Disler, P. |
| Development and Local Contextualization of Mobile Health Messages for Enhancing Disease Management Among Community-Dwelling Stroke Patients in Rural China: Multimethod Study | Gong, E. Y.; Gu, W. B.; Luo, E. D.; Tan, L. W.; Donovan, J.; Sun, C.; Yang, Y.; Zang, L. K.; Bao, P.; Yan, L. J. |
| A mHealth-based care model for improving hypertension control in stroke survivors: Pilot RCT | Lakshminarayanan, K.; Westberg, S.; Northuis, C.; Fuller, C. C.; Ikramuddin, F.; Ezzeddine, M.; Scherber, J.; Speedie, S. |
| Self-medicating practices for managing chronic pain after spinal cord injury | Radwanski, M. |
| Interactive Voice Response-An Innovative Approach to Post-Stroke Depression Self-Management Support | Skolarus, L. E.; Piette, J. D.; Pfeiffer, P. N.; Williams, L. S.; Mackey, J.; Hughes, R.; Morgenstern, L. B. |
| Influence of chain nursing model on quality of life in stroke patients with dysphagia | Tang Chunni, Liao Xilin, Liu Hailan |
| ***No component of self-management (n=4)*** | |
| Pharmacists as Care Providers for Stroke Patients: A Systematic Review | Basaraba, J. E.; Picard, M.; George-Phillips, K.; Mysak, T. |
| Providing systematic detailed information on medication upon hospital discharge as an important step towards improved transitional care | Hohmann, C.; Neumann-Haefelin, T.; Klotz, J. M.; Freidank, A.; Radziwill, R. |
| Investigating the effects of a family-centered care program on stroke patients' adherence to their therapeutic regimens | Nayeri, N. D.; Mohammadi, S.; Razi, S. P.; Kazemnejad, A. |
| PINGS (Phone-Based Intervention Under Nurse Guidance After Stroke) Interim Results of a Pilot Randomized Controlled Trial | Sarfo, F.; Treiber, F.; Gebregziabher, M.; Adamu, S.; Patel, S.; Nichols, M.; Awuah, D.; Sakyi, A.; Adu-Darko, N.; Singh, A.; Tagge, R.; Carolyn, J.; Ovbiagele, B. |
| ***Opinion piece (n=9)*** | |
| Digital health in stroke medicine: what are the opportunities for stroke patients? | Antonenko, K.; Paciaroni, M.; Sokolova, L.; Pezzella, F. R. |
| Antihypertensive medication compliance in African-American stroke patients: Behavioral epidemiology and interventions | Friday, Gary H. |
| Self-monitoring and self-titration of antihypertensive medications result in better systolic blood pressure control | Hung, W. |
| Medication adherence in patients with chronic diseases. Significance and new approaches | Laufs, U. |
| Adherence to secondary prevention strategies after stroke: A review of the literature | Slark, J. |
| A care management intervention improved depression after stroke | Slimmer, L. W. |
| Research progress on application of home rehabilitation nursing for elderly stroke patients in recovery period | Yan Aiying, Qi Lili, Wang Weixin |
| SAFE Medication Management for Patients with Physical Impairments of Stroke, Part One | Yetzer, E.; Blake, K.; Goetsch, N.; Shook, M.; St Paul, M. |
| SAFE Medication Management for Patients With Physical Impairments of Stroke, Part Two | Yetzer, E.; Blake, K.; Goetsch, N.; Shook, M.; St Paul, M. |
| ***Conference abstract (n=47)*** | |
| Collaborative goal-setting and action plans in therapeutic patient education for stroke patients in rehabilitation phase | Accogli, M. A.; Denti, M.; Altavilla, A.; Maisto, G.; Bardelli, R.; Bosi, S.; Cavalli, E.; Pagliacci, D.; Calugi, S.; Costi, S.; Cavazza, S.; Tedeschi, C.; Taricco, M.; Fugazzaro, S. |
| MAAESTRO study-preliminary adherence results of anticoagulated stroke patients | Albert, V.; Polymeris, A. A.; Carco, L.; Hersberger, K. E.; Lyrer, P. A.; Arnet, I. |
| PROTEGE-ACV program: Achieving long-term adherence to secondary stroke prevention goals | Alonzo, C.; Brescacin, L.; Zurru, M. C.; Ortega, F.; Brienza, S.; Montecchia, J.; Gil, D.; Carretero, M.; Ca mera, L.; Cristiano, E.; Waisman, G.; Ovbiagele, B. |
| The role of pharmacists in providing pharmaceutical care in primary and secondary management of stroke: A systematic review | Al-Qahtani, S.; Mason, J.; Paudyal, V.; Jalal, Z. |
| Improving adherence to secondary stroke prevention strategies through motivational interviewing: A randomised controlled trial | Barker-Collo, S.; Barber, P. A.; Witt, E.; Feigin, V. L.; Jones, A.; McPherson, K. |
| A culturally-tailored, skills-based intervention to reduce blood pressure in a multi-ethnic group of mild/moderate stroke survivors with hypertension: Results from the deserve trial | Boden-Albala, B.; Goldmann, E.; Lord, A. S.; Kuczynski, H. M.; Torrico, V.; Appleton, N.; Birkemeier, J.; Turhim, S. |
| A culturally-tailored, skills-based intervention to reduce vascular risk in a multi-ethnic group of mild/moderate stroke survivors: An interim analysis from the DESERVE trial | Boden-Albala, B.; Goldmann, E.; Lord, A. S.; Parikh, N. S.; Kuczynski, H. M.; Tuhrim, S. |
| Shared medical appointments: Supporting stroke care transitions through improved knowledge and peer support | Burns, K.; Theodore, K. S. |
| Medication coaching for secondary prevention adherence in stroke patients: A pilot study | Bushnell, C.; Sides, E.; Zimmer, L.; Olson, D. W.; Pan, W.; Peterson, E. |
| Evaluation of a modified home care intervention for stroke patients in transition from hospital to home | Chen, L.; Sit, J. |
| Chest, Heart & Stroke Scotland Stroke Nurse Service expanding the Service to all patients in Grampian | Chrystal, M.; Sales, F.; Somerville, M.; Macleod, M. J. |
| A questionnaire to assess the feasibility of smartphone applications and wearable devices in consecutive patients attending a London TIA clinic | Clayton, L.; Dados, A.; Simister, R.; Gill, S.; Chandratheva, A. |
| Living well after stroke-piloting a stroke secondary prevention group in the community | Cluckie, G.; Sibson, R.; Bresnai, J. |
| Does a community based, peer support stroke secondary prevention group improve patient's confidence in managing their own health and wellbeing? | Cluckie, G.; Sibson, R.; Corns, J.; Styles, J. |
| The positive impact of the stroke nurse navigator's advocacy on stroke patient's compliance post-discharge | Deen, T.; Terna, T.; Olges, M.; Leahy, B.; Fedder, W. |
| Ulitity of a novel 14 day post stroke unit discharge nurse led telephone clinic to manage modifiable stroke risk factors | Edwards, S.; Macarimban-Inglesant, R.; Simister, R.; Chandratheva, A.; Saber, S. |
| Defining therapeutic patient education in post-stroke rehabilitation: Usual care and preliminary data of an Italian self-management early intervention for stroke survivors (LAY-look after yourself) | Fugazzaro, S.; Bardelli, R.; Accogli, M. A.; Denti, M.; Altavilla, A.; Piccinini, M.; Maisto, G.; Cavalli, E.; Pagliacci, D.; Calugi, S.; Costi, S.; Cavazza, S.; Tedeschi, C.; Taricco, M. |
| A multifaceted pharmacist intervention to support medication adherence after stroke and transient ischemic attack | Hedegaard, U.; Kjeldsen, L. J.; Pottegard, A.; Hallas, J. |
| Comprehensive cardiac rehabilitation feasibility after stroke (CCR FAST) | Herrmann, A.; Chrenka, E.; O'Keefe, L.; Bohnert, B.; House, C.; Nelson, W.; Hanson, L.; Hussein, H. |
| Adapting a team-based secondary stroke prevention intervention in a safety-net setting | Hill, V.; Vickrey, B.; Mittman, B.; Sivers-Texeira, T.; Towfighi, A. |
| Does stroke bridge clinic reduce hospital readmission rates in patients who have suffered a stroke? | Hooker, A. F.; Evans, H. |
| Evaluation of pharmacist clinical interventions and discharge counselling in medical rehabilitation wards | Ip, N. S. R.; Chu, C. K. A.; Chu, L. M. P.; Young, W. M. G.; Tenney, J. W. |
| Does test-enhanced learning improve retention of patient/family stroke education? | Johnson, B.; Urrutia, V. C.; Alexandrov, A. W. |
| Effectiveness of Motivational Interviewing for secondary stroke prevention | Krishnamurthi, R.; Barker-Collo, S.; Witt, E.; Jones, A.; McPherson, K.; Starkey, N.; Parag, V.; Jiang, Y.; Barber, P. A.; Rush, E.; Bennett, D.; Arroll, B.; Feigin, V. |
| Does formal stroke education to inpatients in stroke unit improve their knowledge of stroke? Prospective data from UAE | Kuruvila, J.; Deria, H.; Mathew, S.; Maysoun, E.; Isip, U.; Al Shamisi, S.; Aquino, A.; Altarshi, A.; Canoza, J.; Olidan, J.; Anosa, G.; Renganathan, R. |
| An innovative mHealth-based care model for improving hypertension control in stroke survivors | Lakshminarayan, K.; Westberg, S.; Ezzeddine, M.; Speedie, S.; Fuller, C.; Grimm, R. |
| Development of a collaborative transition coaching program for reduction of post-stroke hospitalizations | Leonhardt, A. M.; Burgen, D. M.; Wolfe, J.; Luther, K. |
| Telephone support program promotes stroke survivor knowledge, self-care, and satisfaction | Lundy, Y. D.; Song, S. Y. |
| Stroke education program improves medication knowledge and satisfaction among stroke patients after hospital discharge | M.W.S, |
| Targets and self-management for the control of blood pressure in stroke and at risk groups (TASMIN-SR): A randomised controlled trial | McManus, R. J.; Mant, J.; Haque, M. S.; Bray, E. P.; Greenfield, S.; Jones, M. I.; Jowett, S.; Little, P.; O'Brien, C.; Penaloza-Ramos, M. C.; Schwartz, C.; Shackleford, H.; Varghese, J.; Williams, B.; Hobbs, F. D. R. |
| Daily goals poster supports effective patient education | Motz, D. S.; Sharp, J. A.; Garcia, T.; Moore, T.; Huey, D.; Austin, T. |
| Overcoming obstacles making people healthy (OOMPH): A patient centered post discharge follow-up program for stroke patients | Muller, J.; Gatton, B.; Fox, L.; Bove, J. A.; Turner, J. D.; Spadaro, M.; Aggarwal, S.; Melville, L.; Melniker, L.; Gaeta, T.; Bova, J.; Salgado, M. |
| Evaluation of a pharmacist-led hypertension-based education program for stroke patients | Nasr, N. B.; Bruandet, M.; Cornillet, N.; Tersen, I.; Rouault, A.; Bezie, Y.; Zuber, M. |
| Pharmacist telephone interventions improve adherence to stroke preventive medications and reduce stroke risk factors: A randomized controlled trial | Nguyen, V. H. V.; Poon, J.; Tokuda, L.; Sayers, J.; Wallis, R. A.; Dergalust, S. |
| Effectiveness of an intervention to improve medication knowledge and adherence in survivors of stroke: A randomised controlled trial | Olaiya, M.; Kim, J.; Nelson, M.; Srikanth, V.; Bladin, C.; Gerraty, R.; Fitzgerald, S.; Phan, T.; Frayne, J.; Cadilhac, D. A.; Thrift, A. G. |
| Virtual care via mobile application using a patient electronic health record portal is feasible and increases stroke patient engagement-a pilot study | Pope, L.; Buchinsky, K.; Noah, P.; Hackett, C.; Cerejo, R.; Tayal, A. H. |
| Does blood pressure self-monitoring with or without supervised antihypertensive treatment modification improve blood pressure (BP) control following stroke or TIA? - The TEST-BP trial | Potter, J. F.; Davison, W.; Clark, A.; Langley, M.; Myint, P. |
| Pharmacist-led transition of care protocol in a primary stroke center: A pilot intervention study | Ramirez, S. T.; Silva-Suarez, G.; Alvarado, Y.; Perez, A. |
| Farmalarm: A mobile application to improve stroke awareness | Requena, M.; Montiel, E.; Rubiera, M.; Muchada, M.; Boned, S.; Molina, C. A.; Ribo, M. |
| Self-monitoring is not just for a study, self-monitoring is for life: An analysis of self-efficacy, blood pressure measurement preference and likelihood to continue monitoring following participation in a self-management study | Schwartz, C.; Koshiaris, C.; Bray, E.; Greenfield, S.; Haque, M.; Hobbs, R.; Little, P.; Mant, J.; Williams, B.; McManus, R. |
| Self-monitoring is not just for a study, selfmonitoring is for life: Analysis of patient likelihood to continue self-monitoring following a hypertension self-management study | Schwartz, C.; Koshiaris, C.; Bray, E.; GreenfiEld, S.; Haque, M.; Hobbs, R.; Little, P.; Mant, J.; Williams, B.; McManus, R. |
| Single-subject analysis of an occupational therapy intervention for medication nonadherence | Schwartz, J. K. |
| Patients' perspectives on the usability of a mobile app for self-management following spinal cord injury | Singh, G.; Mortenson, B.; MacGillivray, M.; Sawatzky, B.; Sadeghi, M.; Mills, P.; Adams, J. |
| The effects of a theory-based health empowerment intervention on self-management and functional recovery post-stroke | Sit, J. W.; Chair, S. Y.; Choi, K. C.; Yip, C. W. H. C.; Ching, R.; Taylor-Piliae, R. E.; Tang, S. W. |
| Uses and Perceptions of mHealth Services Among Post-Stroke Community-Dwelling Smartphone Users and Non-Users | Walsh, R.; Baum, C. M.; Wong, A. |
| Effect of organized stroke education program of stroke unit for acute stroke patients to increase medication compliance after discharge | Yu, H. S.; Jeong, H.; Yoon, N.; Song, H. J.; Kim, J. |
| Research on recurrence rate of ischemic stroke after enhanced management of secondary prevention follow-up intervention | Zhang, J.; Liu, G.; Li, Y.; Wang, B.; Cheng, G. |
| ***Unable to access full-text (n=3)*** | |
| Impact of clinical pharmacist's interventions on health outcomes in post stroke patients | Annie, J.; James, E.; Nambiar, V. |
| Application effect of continuity of care for patients with ischemic stroke | Xie, Ge; Yu, Xiaomeng; Tian, Huijie |
| Application of health management network platform on health management of stroke patients with hypertension after discharge | Zheng, J.; Hu, H. H.; Zheng, X. H.; Li, D. M.; Lu, X. Y.; Zhang, L. J.; Zhang, Y. W.; Yang, P. F.; Li, Q.; Liu, J. M. |
| ***Duplicate (n=1)*** | |
| Feasibility and acceptability of a systemCHANGE(TM) intervention to improve medication adherence in older adult stroke survivors: A pilot randomized controlled trial | Wessol, Jennifer Lynn |
| ***Knowledge syntheses excluded (n=21)*** | |
| Multimodal Interventions to Enhance Adherence to Secondary Preventive Medication after Stroke: A Systematic Review and Meta-Analyses | Al AlShaikh, S.; Quinn, T.; Dunn, W.; Walters, M.; Dawson, J. |
| Mobile apps to improve medication adherence in cardiovascular disease: Systematic review and meta-analysis | Al-Arkee, S.; Mason, J.; Lane, D. A.; Fabritz, L.; Chua, W.; Haque, M. S.; Jalal, Z. |
| A systematic overview of systematic reviews evaluating medication adherence interventions | Anderson, L. J.; Nuckols, T. K.; Coles, C.; Le, M. M.; Schnipper, J. L.; Shane, R.; Jackevicius, C.; Lee, J.; Pevnick, J. M.; Choudhry, N. K.; O'Mahony, D.; Sarkisian, C. |
| Medication Understanding and Taking Self-Efficacy Theory-Based Interventions: A Systematic Review | Appalasamy, J. R.; Ramaiah, S. S.; Quek, K. F.; Zain, A. Z. M.; Tha, K. K. |
| Interventions for improving modifiable risk factor control in the secondary prevention of stroke (Review) | Bridgwood, B.; Lager, K. E.; Mistri, A. K.; Khunti, K.; Wilson, A. D.; Modi, P. |
| Adherence to medication and self-management in stroke patients | Chapman, B.; Bogle, V. |
| Effectiveness, acceptability and usefulness of mobile applications for cardiovascular disease self-management: Systematic review with meta-synthesis of quantitative and qualitative data | Coorey, G. M.; Neubeck, L.; Mulley, J.; Redfern, J. |
| Trials to Improve Blood Pressure Through Adherence to Antihypertensives in Stroke/TIA: Systematic Review and Meta-Analysis | De Simoni, A.; Hardeman, W.; Mant, J.; Farmer, A. J.; Kinmonth, A. L. |
| Self management programmes for quality of life in people with stroke | Fryer, C. E.; Luker, J. A.; McDonnell, M. N.; Hillier, S. L. |
| The role of mHealth for improving medication adherence in patients with cardiovascular disease: a systematic review | Gandapur, Y.; Kianoush, S.; Kelli, H. M.; Misra, S.; Urrea, B.; Blaha, M. J.; Graham, G.; Marvel, F. A.; Martin, S. S. |
| Effect of Mobile Health Interventions on the Secondary Prevention of Cardiovascular Disease: Systematic Review and Meta-analysis | Gandhi, S.; Chen, S.; Hong, L.; Sun, K.; Gong, E. Y.; Li, C. Y.; Yan, L. J. L.; Schwalm, J. D. |
| A systematic review on mobile health applications' education program for patients taking oral anticoagulants | Jang, I. |
| Automated telecommunication interventions to promote adherence to cardio-metabolic medications: Meta-analysis of effectiveness and meta-regression of behaviour change techniques | Kassavou, Aikaterini; Sutton, Stephen |
| Effectiveness of interventions involving nurses in secondary stroke prevention: A systematic review and meta-analysis | Parappilly, B. P.; Field, T. S.; Mortenson, W. B.; Sakakibara, B. M.; Eng, J. J. |
| Effectiveness of mobile applications on medication adherence in adults with chronic diseases: A systematic review and meta-analysis | Peng, Y.; Wang, H.; Fang, Q.; Xie, L.; Shu, L.; Sun, W.; Liu, Q. |
| A Scoping Review of Tailored Self-management Interventions among Adults with Mobility Impairing Neurological and Musculoskeletal Conditions | Plow, M.; Mangal, S.; Geither, K.; Golding, M. |
| The Use of Transitional Care Models in Patients With Stroke | Puhr, M. I.; Thompson, H. J. |
| A Systematic Review and Meta-Analysis on Self-Management for Improving Risk Factor Control in Stroke Patients | Sakakibara, B. M.; Kim, A. J.; Eng, J. J. |
| Evidence-based summary for the safety of multiple medication in elderly patients with ischemic stroke | Tian, X.; Yu, M.; Sun, Y.; Yan, H.; Ma, H.; Jiang, L.; Zhu, Y.; Wang, L.; Ding, Q.; Liu, A. |
| A Systematic Review of Randomized Controlled Trials of Medication Adherence Interventions in Adult Stroke Survivors | Wessol, J. L.; Russell, C. L.; Cheng, A. L. |
| Impact of mobile health and telehealth technology on medication adherence of stroke patients: a systematic review and meta-analysis of randomized controlled trials | Zeng, Z. W.; Wu, T. T.; Lv, M. N.; Qian, J. F.; Chen, M. R.; Fang, Z. W.; Jiang, S. J.; Zhang, J. H. |
